# A phase 1 study of a second experience with Group Retreat Psilocybin Therapy for partial responders after a first experience

**DOI:** 10.3389/fpubh.2026.1810904

**Published:** 2026-04-14

**Authors:** Anthony L. Back, Bonnie A. McGregor, Leslie L. Thorn, Kalin Harvey, Dianna Blom, George Callan, John Guy, Sameet Kumar, Rob Hershberg, Melissa Layer, Jackie Levin, Susanna Myers, Juliana Perez, Kathy Salmonson, Peter Thompson, Joseph Whinney

**Affiliations:** 1 Department of Medicine, University of Washington School of Medicine, Seattle, WA, United States; 2 Fred Hutchinson Cancer Center, Seattle, WA, United States; 3 Orion Center for Integrative Medicine, Seattle, WA, United States; 4 Quantified Citizen, Vancouver, BC, Canada; 5 Harmony Retreats, Union, WA, United States; 6 Private Practice, Seattle, WA, United States; 7 Seattle Mindfulness Center, Seattle, WA, United States; 8 Miami Veterans Affairs Healthcare System, Miami, FL, United States; 9 Independent Researcher, Seattle, WA, United States

**Keywords:** cancer, ceremony and ritual, group, psilocybin, retreat

## Abstract

**Introduction:**

Psilocybin therapy has demonstrated efficacy for cancer-related anxiety and depression, but resource-intensive individual treatment models raise important questions for psychedelic public health about equitable access and scalability. In our prior Phase 1/2 study of group retreat psilocybin therapy for patients with metastatic cancer, we observed partial responders who did not achieve full therapeutic benefit. No published research has examined whether partial responders might benefit from a second psilocybin therapy experience.

**Methods:**

We conducted a single-arm Phase 1 study to assess the safety of a second experience of Group Retreat Psilocybin Therapy for partial responders from our prior study. Protocol modifications addressed dose as a potential contributor to partial response: the initial dose was increased to 35 mg, and an optional 10 mg booster could be requested by participants who reported low subjective effect at 60–90 min and passed a safety check. Pre-retreat antidepressant tapering was not required. The intervention was delivered in a group retreat format with four primary facilitators and included three preparation sessions, a single psilocybin dosing day, and four integration sessions.

**Results:**

Thirteen participants (mean age 56 years, 70% female, 38% on concurrent antidepressants) completed the intervention. No serious adverse events occurred; mild adverse events included transient hypertension (*n* = 4), nausea (*n* = 3), and headache (*n* = 1). Seven participants (54%) received the booster dose. Mean Hospital Anxiety and Depression Scale (HADS) Total scores decreased from 15.08 (SD 4.35) at baseline to 9.00 (SD 4.62) at Day +8, with improvements maintained through 24-week follow-up (mean 10.42, SD 6.93); 69% achieved HADS scores below the clinical threshold. The proportion of participants with a “complete” mystical experience (Mystical Experience Questionnaire ≥ 60%) increased from 38% in the first experience to 77% in the second, without an increase in challenging experiences (Challenging Experiences Questionnaire). Social support, social identification, and group cohesion scores showed progressive improvements that persisted at 24 weeks.

**Discussion:**

A second experience of group retreat psilocybin therapy was safe and feasible for partial responders with metastatic cancer. The protocol modifications—higher dose, optional booster, and no antidepressant tapering requirement—did not introduce new safety concerns and were associated with substantially enhanced mystical experiences and preliminary efficacy signals. These findings support further investigation of retreatment protocols for partial responders and contribute to developing scalable group-based models relevant to psychedelic public health, where the resource intensity of individual treatment remains a fundamental barrier to population-level access.

## Introduction

For patients with metastatic, incurable cancer, unrelieved anxiety and existential distress can cause profound suffering: 25–50% of patients with metastatic cancer experience clinically significant anxiety (1–3) characterized by uncertainty about the future, fear of uncontrollable suffering, feelings of being a burden, loneliness, and grief for the loss of their lives and missed opportunities. Existing treatments are often unsatisfactory (4)—many patients decline or discontinue antidepressants due to emotional blunting (5), and benzodiazepines do not alter the underlying course of anxiety (6).

Psilocybin therapy has emerged as a promising treatment for the anxiety, depression, and existential distress experienced by people living with cancer, but questions of cost and access have yet to be addressed (1, 2, 7). Although multisite trials are in progress, single-site randomized controlled trials using psilocybin therapy conducted using a two-therapist, one-patient model demonstrated impressive efficacy for cancer-related anxiety and existential distress, with effects that are rapid, substantial, and durable (5–7).

While efficacious, this 2-therapist, 1-patient model of psilocybin therapy is resource-intensive, with direct implications for cost and access. The published individual clinical trials required approximately 30 h of direct therapist contact per participant (3, 4, 8), and combined with the projected lack of trained psychedelic therapists (7), these resource issues raise important questions about how psilocybin therapy can be delivered equitably and at scale. Group models offer a promising approach (1, 9). To investigate the group approach to improve access, we developed and tested a group retreat model of psilocybin therapy in a prior Phase 1/2 study (BACK002) (10, 11). In that study with 52 participants over eight retreats, we established the safety of a four-facilitator to eight-participant ratio, with zero instances in which participant distress exceeded the capacity of the facilitation team. Exploratory efficacy analyses revealed clinically and statistically significant improvements in symptoms of anxiety and depression comparable to those observed in 2-therapist, 1-patient trials (10).

However, as in studies using 2-therapist, 1-patient models, we observed some partial responders—participants who did not experience full therapeutic benefit (4, 8). In our group retreat study, partial response manifested in several ways: approximately 40% of participants did not achieve a “complete” mystical experience as measured by the Mystical Experience Questionnaire (MEQ); some participants showed minimal improvement or worsening on the Hospital Anxiety and Depression Scale (HADS); and others retained moderate-to-severe symptoms despite some improvement. Additionally, some participants who initially responded well experienced recurrence of symptoms after the six-month follow-up period. No published research has addressed whether these partial responders might benefit from a second psilocybin therapy experience.

Several factors may contribute to partial responses to psilocybin therapy. First, concurrent or recently tapered antidepressant medications may attenuate the psilocybin effect (12). Evidence from secondary analyses and observational studies suggests that serotonergic antidepressants can diminish both the acute subjective experience and therapeutic outcomes (9–11) although one clinical trial found no significant interaction (12). In our prior study, we observed that participants who had tapered antidepressants reported increased depressive symptoms during the taper period, which seemed counter to our intent to relieve suffering and potentially creating a psychological barrier to fully engaging with the psilocybin experience. Second, the 25 mg dose used in our prior study may have been insufficient for some participants; clinical trials of other conditions have safely used doses up to 40 mg (13, 14), and Oregon's regulated psilocybin program permits doses up to 50 mg. Other factors may include: extent of preparation (no studies have tested the number of preparation sessions (3)); degree of mindfulness capacity (13) (not tested in cancer patients); or proximity to end of life (prior studies included a mixture of stages (4, 8)).

The present study was designed to address these knowledge gaps by offering partial responders from our prior study a second experience of group retreat psilocybin therapy. We made three key modifications to address potential contributors to partial response: (1) participants were not required to taper antidepressants; (2) the starting dose was increased to 35 mg; and (3) a booster dose of 10 mg was made available to participants reporting low subjective effect at 60–90 min after the initial dose. The primary objective was to assess the safety of this second experience. Secondary objectives included assessing the safety of the booster dose and exploring efficacy on symptoms of anxiety and depression.

## Methods

### Patient involvement in trial design

We sought input from participants in our first group retreat psilocybin study as consultants in planning the feasibility and conduct of this study by soliciting brief (approximately 30 min) qualitative interviews specifically on this topic prior to recruitment from 5 participants of BACK002. Participant input was crucial in three design decisions: assessing interest in having a second experience, removing the requirement to taper antidepressants, and extending the integration period from three to four group sessions over a longer time frame.

### Trial design

This was a single-arm Phase 1 study designed to assess the safety of a second psilocybin group retreat experience for patients with metastatic cancer who had participated in and had a partial response to our prior study (BACK002). The study was conducted under FDA Investigational New Drug authorization (IND 165562) and was approved by the Fred Hutchinson Cancer Center Institutional Review Board. The trial was registered at ClinicalTrials.gov (NCT06644170). All participants provided written informed consent.

### Changes to trial protocol

One change was made to the eligibility criteria after initial IRB approval and before recruitment began, based on patient consultant input: we added a fourth criterion allowing participants who had experienced recurrence of anxiety or depression symptoms after completing the six-month follow-up of the first study. No other protocol changes were made during the conduct of the trial.

### Eligibility criteria

Participants were eligible if they had participated in our prior group retreat psilocybin study (BACK002) and met at least one of four partial response criteria: (1) MEQ score below the threshold for a “complete” mystical experience (less than 60% of maximum score); (2) HADS Total change from Day −14 to Day +28 in the first study showing worsening symptoms (defined as increase HADS Total score >2 points) or minimal response (improvement of 5 points or less); (3) HADS Total score at Day +28 in the first study of 11 or greater, indicating persistent moderate-to-severe symptoms; or (4) recurrence of symptoms of anxiety or depression (HADS total score > 11) after completing the six-month follow-up of the first study.

Key inclusion criteria included: diagnosis of metastatic solid tumor or incurable hematologic malignancy; age 18–85 years; ECOG performance status 0–2; HADS Total score of 11 or greater at screening; and ability to participate effectively in a group setting. Key exclusion criteria included: untreated brain metastases; personal or immediate family history of schizophrenia, bipolar disorder, or other primary psychotic disorders; suicidal ideation with Columbia Suicide Severity Rating Scale (C-SSRS) score of 3 or greater; current substance use disorder; QTc interval greater than 450 ms (a risk factor for cardiac arrhythmias); and concurrent use of monoamine oxidase inhibitors or medications that prolong the QTc interval.

Notably, unlike our prior group study, participants were ^*^not^*^ required to taper antidepressant medications and were allowed to continue these medications throughout the study.

### Setting

The study was conducted at a rustic retreat center located approximately 2 h from Seattle, Washington (The Whidbey Institute). This non-clinical setting was selected to provide a comfortable, nature-based environment conducive to the therapeutic process while maintaining all necessary safety provisions, including access to emergency medical services and 24-h on-call cardiology consultation.

### Measures

#### Primary outcome: safety

The primary outcome was the occurrence of adverse events, assessed using a grading system approved by the FDA for psilocybin studies: mild (does not affect patient activity), moderate (mild disruption in usual activity), or severe (major disruption in usual activity). Adverse events were collected using structured questionnaires at the end of the psilocybin session, at 24 h, and at 48 h, with ongoing assessment at all subsequent study contacts. Relationship to treatment was assessed as definite, probable, possible, or unlikely. The FDA-recommended list of abuse-related adverse events for Schedule I substances was used to capture psychological adverse events.

#### Secondary outcome: booster dose safety

Secondary safety outcomes focused on participants who received the booster dose, including blood pressure monitoring at 1 h post-booster; if that BP was high, it was monitored hourly until < 150/90.

#### Exploratory efficacy outcomes

The primary efficacy measure was the Hospital Anxiety and Depression Scale (HADS) Total score, assessed at Day −14 (baseline), Day +7, Day +21, Day +35, Week 8, Week 12, and Week 24. The HADS is a 14-item self-report measure with separate subscales for anxiety (HADS-A) and depression (HADS-D); total scores range from 0 to 42, with higher scores indicating greater symptom severity (14). The HADS was chosen because it was used in prior studies of psilocybin therapy for cancer, has been extensively validated in cancer patients, and avoids somatic symptoms that could overlap with cancer-related fatigue. A decrease in HADS scores indicates improvement.

Other exploratory measures included: the Demoralization Scale II (DS-II) is a 16-item self-report instrument designed to assess demoralization (15, 16); each item is rated on a 3-point Likert scale, with total scores ranging from 0 to 32, where higher scores indicate greater demoralization. The Death and Dying Distress Scale (DADDS) is a 15-item measure used to assess distress related to thoughts of death and dying (17); each item is rated on a 6-point Likert scale, with total scores ranging from 0 to 75, where higher scores indicate greater distress. The Adjustment Disorder New Module (ADNM-20) is a 20-item self-report measure designed to assess adjustment disorder symptoms according to ICD-11 criteria; each item is rated on a 4-point Likert scale (1 = never to 4 = frequently), with total scores ranging from 20 to 80, where higher scores indicate greater adjustment disorder symptomatology (18). The National Institutes of Health Healing Experience of All Life Stressors (NIH-HEALS) was used to assess psychosocial and spiritual healing (19); each item is rated on a 5-point Likert scale, with total scores ranging from 35 to 175, where higher scores indicate greater wellbeing. The Functional Assessment of Cancer Therapy–General (FACT-G) is a 27-item measure used to assess quality of life that has been widely used in cancer studies (20). Each item is scored on a 5-point Likert scale, with scores ranging from 0–108, and higher scores indicate better functioning.

We added process measures not used in our prior study to assess group experience and social outcomes: Strength of Drug Effect was measured using a 2-item visual analog scale assessing the intensity of the subjective psilocybin experience at 60–90 min post-dose, administered only to participants who requested evaluation for a booster dose. Social Identification was assessed with a single item asking participants to rate agreement with the statement “I identify with [this retreat group]” on a 7-point scale, a validated approach for measuring group identification (21). Social Support was measured using an 8-item scale assessing the perception of emotional, instrumental, and informational support, with items rated on a 5-point frequency scale (22). Purpose and Meaning was assessed with a 5-item scale adapted from the Purpose in Life subscale of the Ryff Scales of Psychological WellBeing, measuring the extent to which participants feel their lives have meaning and direction (23). Global Impression of Change was measured with a single item asking participants to rate their overall change since baseline on a 7-point scale ranging from “very much improved” to “very much worse” (24).

The aforementioned measures were collected on a secure mobile app (Quantified Citizen).

Additional on-site measures addressing aspects of the psilocybin session were collected on paper on the morning of Day 3 at the retreat: Mystical Experience Questionnaire 30 (MEQ30), a 30-item self-report measure with scores from 0–150, with higher scores indicating greater mystical experience intensity (25); the Challenging Experience Questionnaire, a 26-item self-report measure with scores from 0–130 with higher scores indicating more challenging experiences (26); the Emotional Breakthrough Inventory (EBI), a 6-item measure with scores from 0–600, with higher scores indicating more profound emotional breakthroughs (27); and the Communitas scale, a 10-item measure with scores of 10–70 with higher scores indicating stronger group bonding (28).

### Intervention

**Figure 1:**
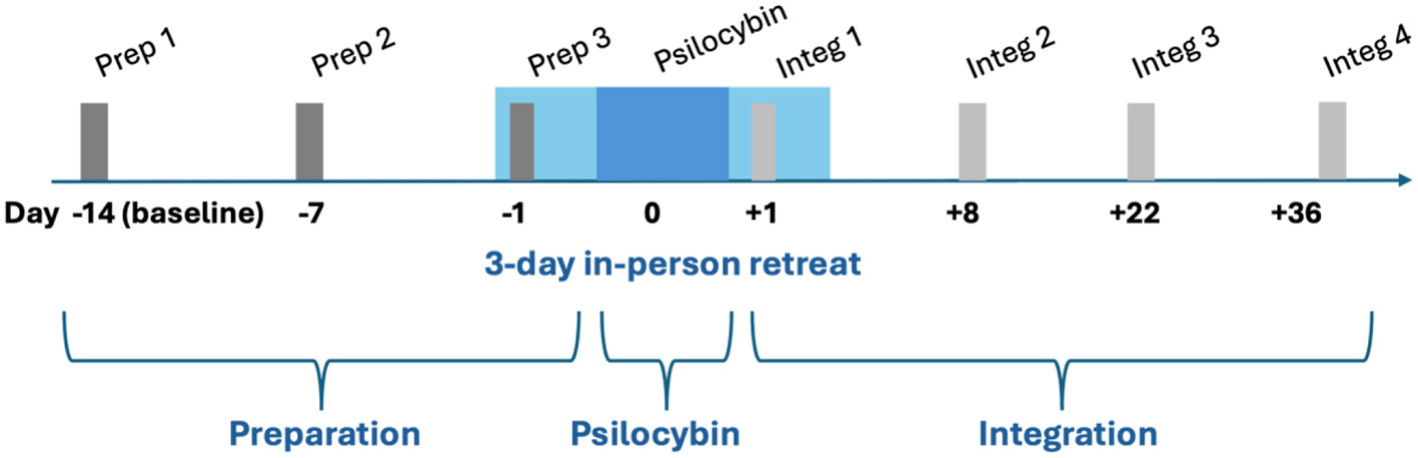
Intervention diagram. The 3 preparation sessions, all 90 min, are shown by dark gray bars. Preparation sessions 1 and 2 were virtual. The 4 integration sessions, all 90 min, are shown by light gray bars. Integration sessions 2, 3, and 4 were virtual.

The intervention consisted of group retreat psilocybin therapy delivered over a three-day retreat, embedded within a structured program of preparation and integration sessions (Figure 1). Detailed descriptions have been published elsewhere (10, 11). The Reporting of Setting in Psychedelic Clinical Trials (ReSPCT) checklist for reporting set and setting is included in the Supplementary material (29).

#### Preparation phase

Participants completed three 90-min group preparation sessions. The first two sessions were conducted via videoconference at approximately Day −14 and Day −7. The third preparation session was conducted in person at the retreat center on Day −1. These sessions focused on building group cohesion, establishing therapeutic rapport, setting intentions, and preparing participants for the psilocybin experience. Participants also completed one individual preparation session in person on Day −1.

#### Psilocybin session

On Day 0, participants received psilocybin PEX010 (Filament Health, Vancouver, BC), a partially purified botanical extract of Psilocybe cubensis manufactured under cGMP conditions. The starting dose was 35 mg psilocybin, administered orally in capsules with lukewarm lemon ginger tea.

A booster dose of 10 mg psilocybin was available to participants who reported low subjective drug effect at 60–90 min after the initial dose. Prior to administering the booster, participants underwent a safety assessment including: ability to walk without assistance to the evaluation station; blood pressure less than 150/90 mm Hg; temperature less than 38 C; physical examination for signs of serotonin syndrome using Hunter criteria; and 12-lead electrocardiogram with QTc less than 450 ms. The ECG was done in a private room since it required disrobing, and took about 10 min using an ECG with a single-use, pre-positioned set of leads (QT Medical, Diamond Bar CA). Participants who met all safety criteria and reported low subjective effect were offered the booster dose. No participant received more than 45 mg total psilocybin.

The psilocybin session was conducted in a group setting with four core facilitators and two backup facilitators, with a prespecified maximum of eight participants (10). The facilitation team included two lead facilitators (licensed healthcare providers with specialized training in psychedelic therapy) and two associate facilitators. A physician was present on-site throughout the retreat. Participants remained under observation until the facilitation team judged their perception, cognition, and functioning adequate for them to retire for the evening. A facilitator and the physician were available on-call throughout the 3-day retreat for any concerns.

#### Integration phase

Participants completed four 90-min group integration sessions, an increase from three sessions in the prior study. The first integration session was conducted in person at the retreat center on Day +1. Subsequent sessions were conducted via videoconference at Week 1, Week 3, and Week 5 after leaving the retreat. Participants also completed one individual integration session in person on Day +1. This extended integration period was implemented in response to observations from the prior study that participants benefited from longer-term support.

### Outcomes

#### Statistical methods

This Phase 1 study was designed primarily to assess safety and preliminary efficacy to guide power calculations for future studies. Given the number of participants who met criteria for inclusion, we estimated enrollment of up to 16 participants in two retreats.

Descriptive statistics were generated for all safety outcomes. Given the small sample size and exploratory nature of efficacy measures, only descriptive statistics were generated, no other tests were performed. Data are presented in tables and graphs [Tableau Cloud (2025.2)].

## Results

### Participant flow

**Figure 2:**
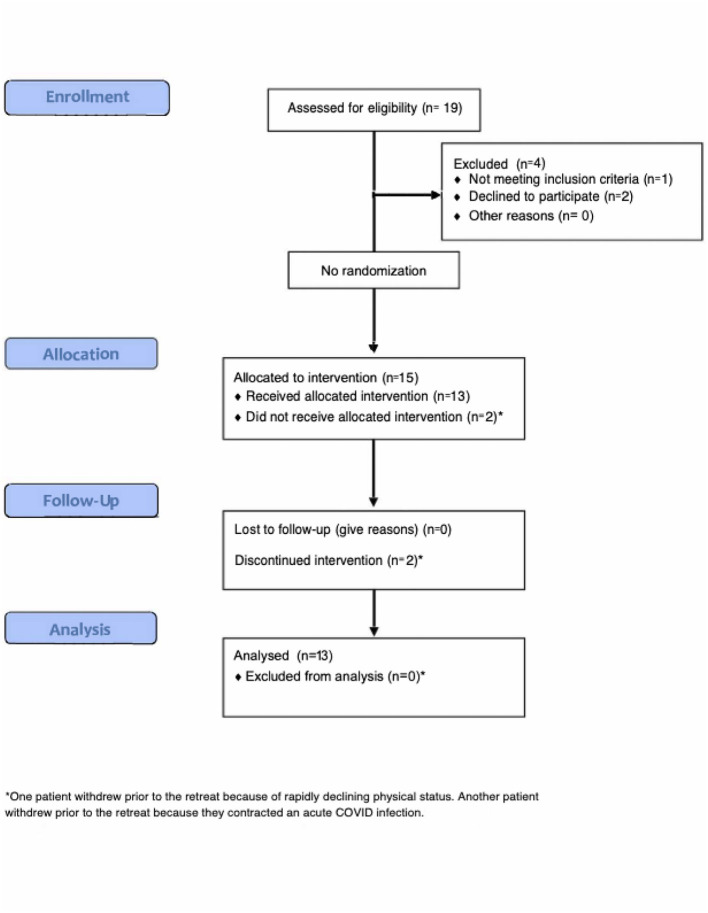
CONSORT diagram.

Of the 52 participants who completed the prior study (BACK002), 19 met at least one partial response criterion and were potentially eligible. Of these, 19 expressed preliminary interest in participating, 19 were screened, and 15 were enrolled. The average time from their first psilocybin session in BACK002 to their second psilocybin session in BACK004 was 15.3 months (range 10–20 months). The CONSORT diagram is in Figure 2, Of the 15 enrolled participants, one participant developed acute COVID-19 infection prior to the retreat and was unable to attend, and one other participant experienced a rapidly declining functional status after enrollment and was unable to attend. Consequently, thirteen participants attended a three day retreat, completed the psilocybin session and entered the integration phase. Twelve of 13 treated participants completed all study assessments through the 24-week follow-up (Figure 2), except for 1 participant who had medical complications precluding her Month 2 questionnaires, and died of progressive cancer prior to the Month 6 questionnaires.

### Recruitment

Recruitment occurred from October 2024 through March 2025. The retreats were conducted in February and March 2025. Follow-up questionnaires were completed in October 2025.

### Baseline characteristics

Table 1 presents baseline demographic and clinical characteristics of the participants who attended a retreat. These 13 participants met the partial response criteria as follows: 9 participants met Criterion 1 (MEQ below complete threshold); 0 met Criterion 2 (minimal or negative HADS change); 1 met Criterion 3 (HADS > 11 at Day +28); and 3 met Criterion 4 (symptom recurrence after 6-month follow-up).

**Table 1:** Demographics.

Characteristic	All participants (*n* = 13)
	**N** (%)
Age	
years mean (range)	56 (33, 66)
Sex	
Female	9 (70%)
Male	4 (30%)
Ethnicity	
White	13 (100%)
Hispanic	1 (8%)
Marital status	
Married	9 (70%)
Partnered	1 (7%)
Single	3 (23%)
Type of cancer	
Breast	3 (23%)
Gynecological	3 (23%)
Gastrointestinal	4 (31%)
Other (sarcoma, thyroid, myeloma)	3 (23%)
Anticancer therapy during intervention	
Yes	11 (85%)
No	2 (15%)
Duration living with cancer at study entry	
Months mean (range)	47 (18, 87)
Employed	
Yes	7 (54%)
No	6 (46%)
Antidepressants (not tapered)	
Yes ^*^	5 (38%)
No	8 (62%)

^*^Bupropion (4), Duloxetine (1).

Their mean age was 56 years (range 33–66); 9 (70%) were female. All participants identified as White, with 1 (8%) identifying as Hispanic. Cancer types included gastrointestinal (31%), breast (23%), gynecological (23%), and other (sarcoma, thyroid, myeloma; 23%). Mean duration living with cancer was 47 months (range 18–87). Eleven participants (85%) were receiving active anticancer therapy during the study period.

Five participants (38%) were taking antidepressant medications (bupropion, *n* = 4; duloxetine, *n* = 1) and continued these medications throughout the study without tapering. These five participants had all been taking the same antidepressant prior to BACK002 (the prior study), had tapered for that first psilocybin session, and resumed taking them during the integration period.

### Intervention delivery

All 13 participants completed the three preparation sessions and received the 35 mg psilocybin dose. Seven participants (54%) received the 10 mg booster dose after meeting safety criteria and reporting low subjective drug effect at 60–90 min. Of these seven, 3 were taking concurrent antidepressants. All participants completed the four group integration sessions.

### Primary outcome: safety

No serious adverse events occurred. No moderate or severe adverse events attributable to psilocybin were observed. There were no instances of suicidal ideation, psychotic symptoms, or prolonged psychological distress requiring pharmacological intervention.

Mild adverse events for participants who attended the retreat are summarized in Table 2. Four participants experienced transient asymptomatic hypertension (blood pressure elevated above baseline but below 150/90 mm Hg). Three participants experienced nausea (mild in two, moderate in one; the participant with moderate nausea received ondansetron). One participant experienced self-limited somatic shaking during the session. One participant with a history of migraines experienced a mild headache at the end of the dosing session and self-administered their own rizatriptan. One participant, who had attended a family funeral the day before the retreat, experienced a prolonged but uncomplicated drug effect; they remained recumbent at the end of the dosing session but were responsive and oriented, participated in dinner, retired normally, and reported feeling well the following morning.

**Table 2:** Safety outcomes.

Adverse event	Number of participants	Severity	Comments	Relationship to booster?
Hypertension	4	Mild	All asymptomatic	1 occurred after booster
Nausea	3	Mild 2, Moderate 1	1 received ondansetron	Moderate occurred after booster
Somatic reaction	1	Mild	Self-limited shaking during session	
Headache	1	Mild	History of migranes, took own rizatriptan	Took booster, headache occurred at very end of dosing session
Prolonged drug effect	1	Mild	Came to retreat exhausted from uncle's funeral prior day.	Took booster, still lying on mat at end of session, but responsive and oriented. Got up for dinner, went to bed, had check 1.5 h later, next morning felt ok.
Acute COVID infection	1	Mild	Acquired prior to retreat, did not come, assessed by oncology team, treated with Paxlovid	

### Secondary outcome: booster dose safety

All seven participants who received the booster dose tolerated it without serious or moderate adverse events. Among these seven participants, one experienced asymptomatic hypertension post-booster, one experienced moderate nausea, one experienced a mild headache, and one experienced prolonged drug effect (all described above). Table 3 presents QTc interval changes from screening to post-initial-dose assessment (just prior to booster administration). Mean QTc change was +1 ms (range −29 to +26 ms). After their initial psilocybin dose, no participant who wanted to be assessed for a booster had QTc prolongation above 450 ms that would have precluded booster administration. Two of the participants who received a booster were taking bupropion; one participant who received a booster was taking duloxetine.

**Table 3:** QTc changes.

Screening QTC interval	Booster QTC interval	Change in QTc 2nd minus 1st
409	420	+11
468	450	−18
434	437	+3
422	448	+26
420	391	−29
418	437	+19
430	419	−11

The mean difference for the change in QTc from the screening visit to after the initial psilocybin dose just before the booster dose was 1 ms.

### Exploratory efficacy outcomes

#### Anxiety and depression

**Figure 3:**
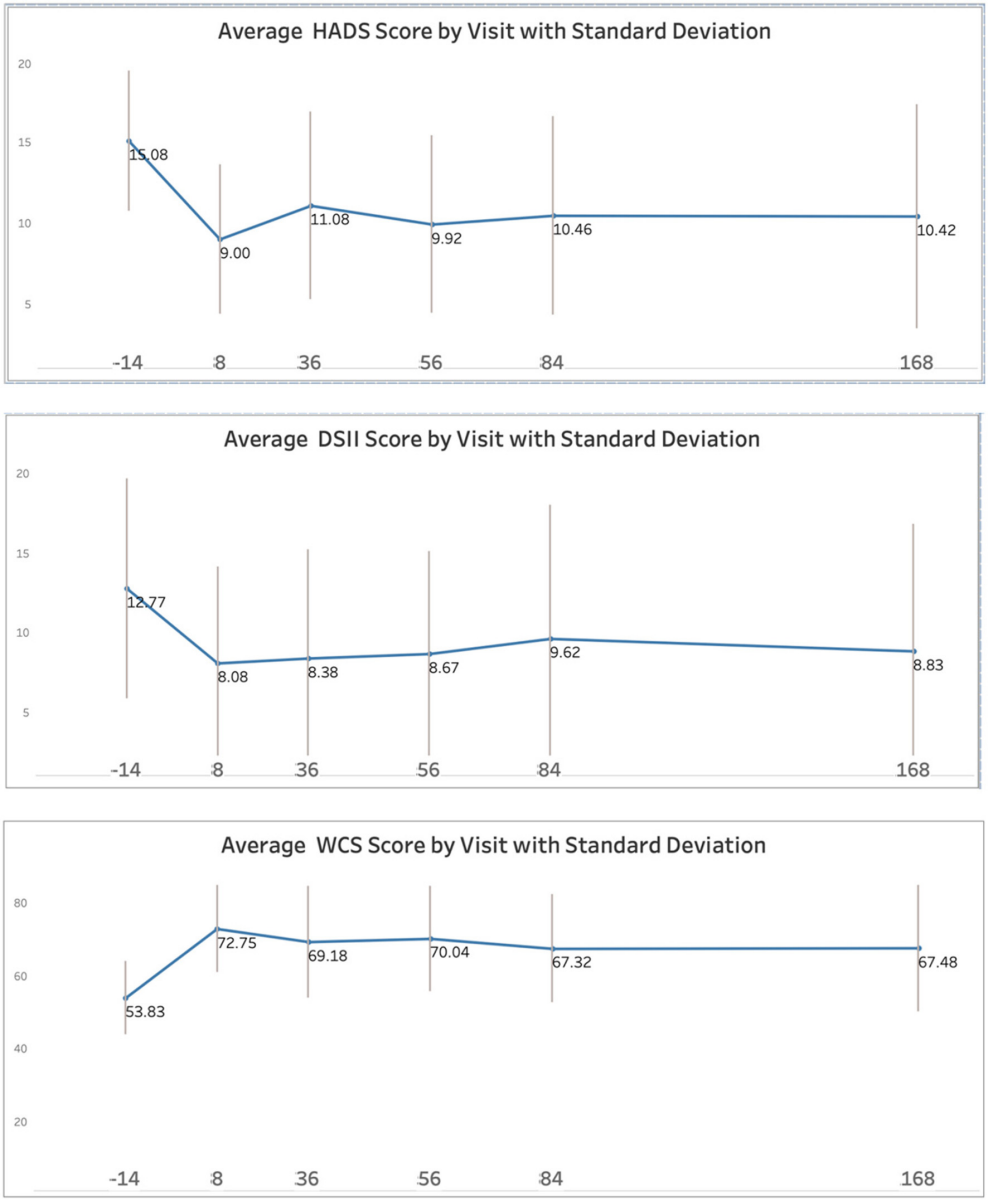
Exploratory outcomes (Figures). HADS, Hospital Anxiety and Depression Scale; DSII, Demoralization II Scale; WCS, Watts Connectedness Scale. The Y axis shows the questionnaire score; the X axis shows the Day of study timepoint, where −14 = baseline and +8 = Day +8 after the psilocybin session. The vertical lines display standard deviation at each timepoint.

HADS Total scores decreased substantially from baseline to Day +8, with improvements maintained through the 24-week follow-up (Table 4; Figure 3). Nine participants (69%) had reduction in symptoms to “mild,” defined as HADS Total ≤ 11, at Day +8. Two participants experienced 6-point increases in HADS scores between Day +8 and Day +36 attributable to external life stressors unrelated to their cancer: one was managing a close relative's estate amid high family conflict; another began peer counseling other cancer patients with minimal support, taking on their distress.

**Table 4:** Psychological and social outcomes.

Measure	D−14 (Baseline)	D+8	D+168	Direction
Psychological outcomes, mean (SD)				
HADS total	15.08 (4.35)	9.00 (4.62)	10.42 (6.93)	Improved
FACT-G (Quality of Life)	63.77 (15.18)	73.92 (16.36)	73.17 (20.82)	Improved
DS-II (Demoralization)	12.77 (6.91)	8.08 (6.08)	8.83 (7.98)	Improved
DADDS (Death Anxiety)	29.38 (16.51)	21.00 (16.35)	18.58 (15.38)	Improved
NIH-HEALS	119.38 (15.40)	128.46 (17.53)	126.00 (22.29)	Improved
ADNM-20 (Adjustment)	53.31 (7.33)	45.23 (11.19)	46.50 (16.16)	Improved
Watts Connectedness	53.83 (10.04)	72.75 (11.85)	67.48 (17.26)	Improved
Social and group outcomes				
Social support	37.80 (13.08)	50.77 (5.61)	50.25 (6.09)	Improved
Social identification	4.17 (1.47)	6.09 (0.86)	5.92 (1.16)	Improved
Purpose and meaning	17.67 (2.25)	19.23 (3.59)	18.92 (4.60)	Improved
Group cohesion^*^	8.30 (1.21)	8.85 (0.38)	8.83 (0.39)	Improved

^*^Group Cohesion baseline measured at D−1 (after 2 preparation sessions). HADS, Hospital Anxiety and Depression Scale; FACT-G, Functional Assessment of Cancer Therapy-General; DS-II, Demoralization Scale II; DADDS, Death and Dying Distress Scale; NIH-HEALS, National Institutes of Health Healing Experience of All Life Stressors; ADNM-20, Adjustment Disorder New Module.

#### Other psychological outcomes

All psychological outcome measures showed improvement from baseline to Day +8, with gains largely maintained at 24-week follow-up (Table 4; Figure 3). Quality of life (FACT-G) improved by over 10 points, while demoralization (DS-II) and death anxiety (DADDS) decreased. Psychosocial functioning (NIH-HEALS), adjustment (ADNM-20), and feelings of connectedness (Watts Connectedness Scale) all improved (Table 4).

#### Social and group outcomes

**Figure 4:**
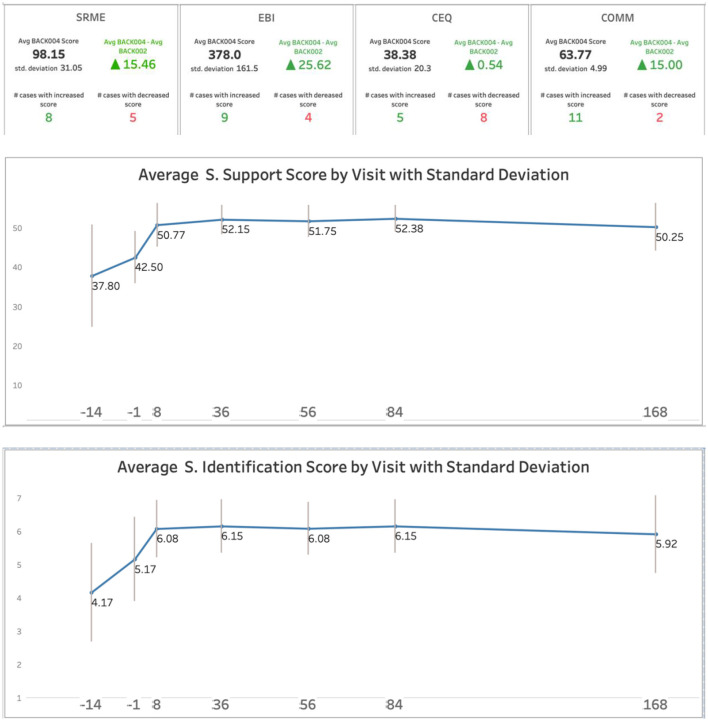
Process & Social measures. SRME, Standard (MEQ30) Mystical Experience Questionnaire; EBI, Emotional Breakthrough Inventory; CEQ, Challenging Experiences Questionnaire; COMM, Communitas Scale; S. Support, Social Support Scale; S. Identifcation, Social Identification Scale. Black, 2^nd^ experience scores; Green, Change compared with 1^st^ experience for the same participants.

Social process measures showed progressive improvements across the intervention period (Table 4; Figure 4). Social support and social identification increased from baseline through Day +8 and were maintained at 24 weeks. Group cohesion, measured from Day −1 onward, was high throughout and increased slightly after the psilocybin session. Purpose and meaning scores showed modest improvements. None of these measures were collected in the first experience study, precluding direct comparison.

#### Psilocybin session experience

**Figure 5:**
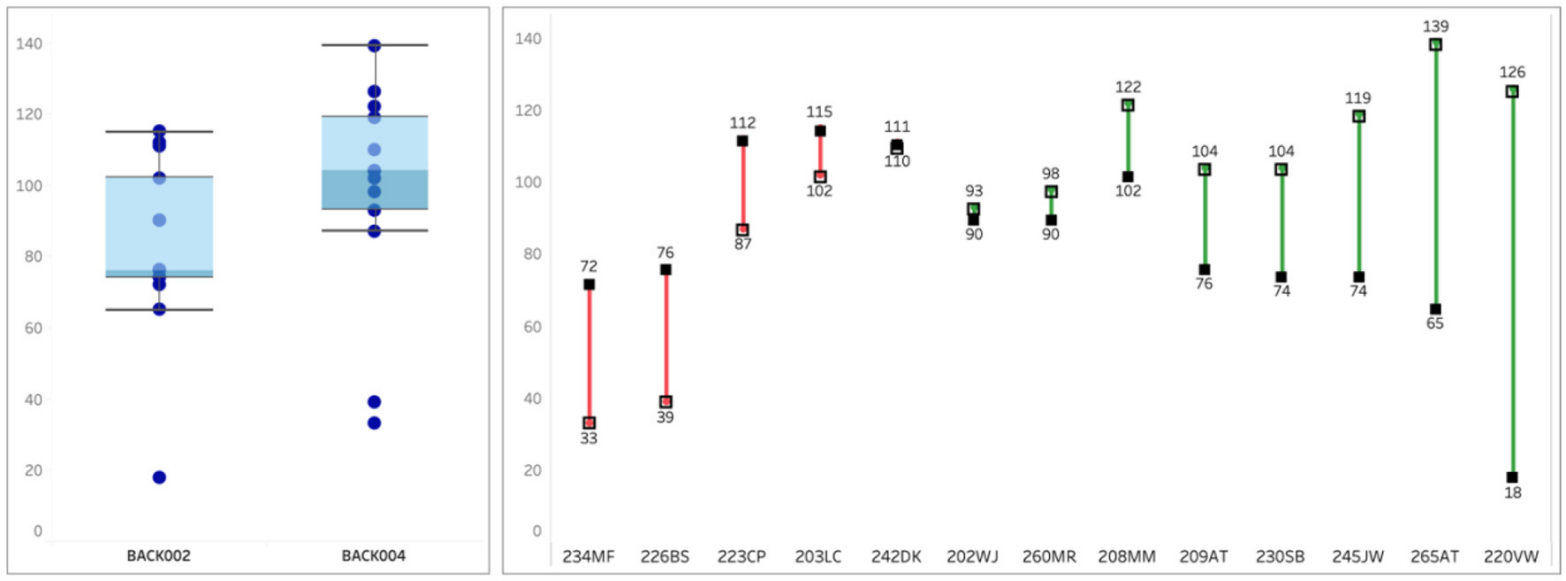
Comparisons 1^st^ experience vs. second experience, Mystical experience questionnaire. On the left: a box plot of MEQ30 scores from the participants in this trial from their first experience **(left)** and second experience **(right)**. On the left panel, the box plot shows the median MEQ score (line between the light blue and dark blue boxes), the interquartile range (25th percentile at bottom of darker blue box; 75th percentile at top of lighter blue box), whiskers at 1.5x the interquartile range, and outliers. On the right: each participant's first experience score (solid dot) and second experience score (open dot); scores are arranged from participants whose second experience scores were lower than their first experience (red lines) to participants whose second experience scores were higher than their first experience (green lines).

Mystical experience scores were substantially higher in the second experience compared to participants' first experience (Figure 5). The proportion achieving a “complete” mystical experience (MEQ ≥ 60%) doubled from 38% to 77%. Emotional Breakthrough Inventory and Communitas scores also increased, with 9 of 13 and 11 of 13 participants showing increases, respectively (Figure 4). Notably, Challenging Experience Questionnaire scores remained essentially unchanged, indicating that the enhanced mystical experience did not come at the cost of increased psychological difficulty (Figure 4).

#### Participant views

No participant regretted coming for a second experience; in fact, all felt the second experience was valuable, and built on the first experience. One participant reported:

“My first one was this very emotional experience, and kind of opened the emotional realm for me. My second experience had more spiritual elements...I've used the term spiritual just because I don't have better words for it, a feeling of being interconnected…a sense of something beyond “this mortal coil”...But the second one couldn't have happened without the first one, right?”

Another reported:

“The first one was foundational, heart opening, expansive…it was like validation… just to feel so loved, cared for, it set this huge foundation. The second one I could go deeper into things…like the pain, the shame, the fear….tap into that unit of consciousness that coming in, thinking about this craziness of the human experience. And I was sharing [to my spouse] how I was just saying, well, maybe we are not here to have just the happy ever after endings….life contains the good, the bad, the ugly. And the blissful and the wonderful, right?”

## Discussion

This Phase 1 study is the first to examine whether metastatic cancer patients with moderate to severe symptoms of anxiety and depression who were partial responders to a first experience with psilocybin therapy can benefit from a second experience with acceptable toxicity and preliminary signals of clinical benefit. Among 13 partial responders to a first experience with Group Retreat Psilocybin Therapy, a second experience was safe and well-tolerated even with a higher initial psilocybin dose of 35 mg and an optional booster dose of 10 mg. No participant had a serious adverse event.

### Safety of a second experience at higher doses

The safety profile observed in this study was reassuring and consistent with our prior study and the broader psilocybin literature. No participants experienced serious adverse events, psychotic symptoms, suicidal ideation, or psychological distress requiring pharmacological intervention. The adverse events that did occur—transient hypertension, nausea, headache, and one prolonged but uncomplicated drug effect—were mild and self-limited, consistent with the known acute effects of psilocybin (15, 16).

The higher starting dose of 35 mg and the availability of a 10 mg booster dose did not introduce new safety concerns. Seven participants (54%) received the booster dose, and all tolerated it well. The safety screening protocol—requiring ambulation, acceptable vital signs, absence of serotonin syndrome signs, and QTc less than 450 ms—proved practical to implement in the group retreat setting. Mean QTc change at the time of booster assessment was not clinically significant at only +1 ms, and no participant was excluded from receiving the booster due to QTc prolongation. These findings support the feasibility of individualized booster dosing within a group treatment model, and suggest that a QTc measurement at the time of booster administration is unnecessary.

### Feasibility of maintaining antidepressant medications

A notable feature of this study was that participants were not required to taper antidepressant medications—a departure from most psilocybin clinical trials. Five participants (38%) continued their antidepressants throughout the study, and there were no safety concerns in this subgroup. This finding is clinically important because in our prior study, antidepressant tapering resulted in increased symptom burden prior to the psilocybin session for at least 3 participants. In addition, participants who had tapered antidepressants had a non-significant trend toward reduced MEQ scores and poorer HADS outcomes. Thus for the present study, we hypothesized that the combination of a higher dose, an available booster, and no tapering requirement might yield better outcomes than tapering followed by a lower dose.

### Enhanced mystical experience in the second session

The most striking finding may be the substantial increase in mystical experience scores from the first to second psilocybin session. Mean MEQ scores increased from 77 to 107, and the proportion achieving a complete mystical experience (≥60% of maximum score) increased from 38% to 77%. This improvement occurred despite—or perhaps because of—the fact that these participants were specifically selected as partial responders to their first experience.

Notably, 9 of the 13 participants were eligible for this second experience study because of a low MEQ score at their first experience, but in their second experience, their MEQ scores increased from a mean of 76.0 in their first experience to mean 98.15 in their second experience. The proportion of participants whose MEQ scores qualified as a “complete” mystical experience doubled from 38% in their first experience to 77% in their second experience. These results suggest that for partial responders after a first experience, a second experience appears to allow them to have a more “mystical” experience, which has been associated with lasting benefit.

Several factors may have contributed to this enhancement. The higher starting dose (35 mg vs. 25 mg) and availability of a booster dose likely increased the pharmacological intensity of the experience for some participants. The absence of antidepressant tapering may have allowed participants to enter the experience with a more stable psychological baseline. Participants' prior experience with the group retreat format may have reduced anticipatory anxiety and allowed greater psychological surrender to the experience. Finally, the established relationships that participants had with the facilitation team may have created an especially supportive interpersonal context, even though they were in different cohorts for their first experience.

Importantly, the increase in mystical experience intensity was not accompanied by an increase in challenging experiences. Mean Challenging Experiences Questionnaire scores were nearly identical between the first and second experiences, suggesting that the enhanced mystical experience did not come at the cost of increased psychological difficulty.

### Exploratory efficacy signals

The exploratory efficacy outcomes were encouraging. Although this Phase 1 study was not powered to detect efficacy, the observed improvements in symptoms of anxiety and depression are noteworthy. The HADS Total scores decreased by 7 points at Day +8, with this improvement maintained through the 24 week followup. Since the minimal clinically important difference in the HADS was 1.7 in a study of patients with serious illness (cardiovascular) (30), this improvement is clinically significant. In addition, the 7-point reduction in HADS Total scores is comparable in magnitude to effects observed for a first psilocybin therapy experience in randomized controlled trials of individual psilocybin therapy for cancer-related distress (5–7). In this second experience study, 69% of participants achieved HADS Total scores below 11—the threshold for clinically significant symptoms—at Day +8.

The trajectory of HADS scores revealed some complexity. Two participants experienced increases in HADS scores between Day +8 and Day +36 that were attributable to external life stressors unrelated to their own cancer—one participant was dealing with grief from a close relative's death; another had been invited to become a peer counselor for other patients with little instruction and experienced distress that they attributed to the responsibility of this unfamiliar role. These cases illustrate that the stresses of living with advanced cancer do not occur in isolation, and that people living with cancer face a succession of challenges.

Measures of death anxiety (DADDS), demoralization (DSII), quality of life (FACT), and adjustment disorder (ADNM20) changed, on average, in the direction of clinical improvement. We did not test these changes statistically because this was a small single-arm safety study. However, these scores may not tell the entire story; even when individual quantitative scores suggested no change or even negative, every participant felt that their second experience was a valuable continuation of their first experience in their ongoing psychospiritual adjustment to living with cancer.

### Social and group processes

Measures of social processes in this study also provide preliminary evidence that some benefits of the group retreat model are the result of enhanced social connection and group cohesion. Social support scores increased progressively from baseline through Day +8 and were maintained at 24 weeks. Social identification—the sense of belonging to and identifying with the group—showed a similar pattern. Group cohesion scores, not measured at the pre-Prep 1 baseline, were measured from Day −1 onward, were high throughout, increasing slightly after the psilocybin session.

These social process measures were not collected in the first experience study, so direct comparison is not possible. However, the Communitas Scale—which assesses the sense of an experience of community during the psilocybin session—showed substantially higher scores in the second experience compared to the first (mean increase of 15 points, with 11 of 13 participants showing increases). Further research is needed to examine the how preparation and retreat design may enhance the power of social processes in group psilocybin therapy.

### Implications for research and clinical practice

These findings have several implications for the developing field of psilocybin therapy. First, they suggest that partial response to a single psilocybin therapy session need not be considered a psilocybin therapy treatment failure. Psilocybin therapy may benefit from dose optimization and retreatment for some patients.

Second, the safety of higher doses and booster dosing in a group setting suggests that individualized dosing protocols may be feasible in future clinical implementations. The safety screening protocol used in this study—practical enough to implement during a group retreat—could serve as a model for clinical programs seeking to optimize dosing for individual patients.

Third, the feasibility of maintaining antidepressant medications has important implications for access. Many patients with cancer-related distress are already taking antidepressants, and requiring tapering creates a significant barrier—both logistically and because of the distress that tapering can cause. If future studies confirm that beneficial outcomes can be achieved with appropriate psilocybin dose adjustments rather than tapering of antidepressants, this may reduce a barrier to psilocybin therapy.

Fourth, the enhanced social outcomes observed in this study support continued investigation of group treatment models. If the group retreat format provides therapeutic benefits beyond those of individual treatment—through enhanced social connection, shared meaning-making, and mutual support—this could help justify the additional complexity of group logistics while substantially improving cost-effectiveness and access.

### Limitations

This study has several important limitations. The single-arm design without a control group precludes causal inference about efficacy. The small sample size limits statistical power and precision of effect estimates, and precludes meaningful subgroup analyses (e.g., comparing outcomes in those who did vs. did not receive the booster, or those on vs. off antidepressants).

The study population was highly selected: all participants had previously completed a psilocybin therapy study, were motivated to have a second experience, and had demonstrated ability to participate effectively in a group format. The lack of racial, and ethnic diversity (all participants identified as White) limits generalizability to broader populations and reflects ongoing challenges in ensuring equitable access to psychedelic research.

This second experience protocol, while tailored to these participants, included multiple protocol changes (dose, booster availability, no tapering requirement, extended integration), so attributing improvements to any single factor is not possible.

Finally, we note that this study was conducted by investigators with extensive experience in psilocybin therapy and at a retreat center purpose-designed for this work. Whether similar safety and efficacy could be achieved by less experienced teams or in less carefully controlled settings is untested.

### Relevance to psychedelic public health

This study speaks directly to several priorities articulated in the emerging field of psychedelic public health. First, regarding models of psychedelic care within public health systems, this Group Retreat Psilocybin Therapy model addresses a fundamental barrier to population-level access: the resource intensity of individual treatment. By demonstrating safety with a four-person facilitator team for second experience retreats with 6 and 7 participants—and now with higher doses and individualized booster dosing—this work contributes to developing scalable models that could realistically be integrated into public health infrastructure. The workforce implications are substantial: if equivalent outcomes can be achieved with group models requiring one-quarter the clinician hours per participant, psilocybin therapy becomes far more feasible as a public health intervention for the estimated 25–50% of patients with advanced cancer who experience clinically significant distress.

Second, this study exemplifies the integration of spirituality, ceremony, and public health services. The retreat setting, facilitation model that treats the intervention as a rite of passage, the emphasis on group cohesion and communitas, the use of mystical experience as both outcome and therapeutic mechanism all reflect an approach that honors the ceremonial dimensions of psychedelic healing within a rigorous clinical research framework. The progressive increases in social identification, group cohesion, and communitas scores suggest that the group format may facilitate relational and transpersonal experiences that contribute to therapeutic benefit.

Third, this work offers a critical perspective on medicalization and sustainability. Rather than pursuing ever-more-intensive individualized medical interventions, the group retreat model asks whether healing can emerge from community, shared experience, and mutual support—resources that are inherently renewable rather than scarce (31). The involvement of prior participants as consultants in trial design further reflects a participatory ethos often not seen in medically focused, top-down trial designs. Finally, as a study addressing existential distress in patients with incurable cancer, this work contributes directly to psychedelic applications for cancer-related anxiety, depression, and existential distress, where the need for innovative, accessible approaches is acute.

## Conclusions

In this Phase 1 study, a second experience of group retreat psilocybin therapy for partial responders with metastatic cancer was safe and feasible. The protocol modifications—higher starting dose, optional booster, and no antidepressant tapering requirement—did not introduce new safety concerns and were associated with substantial improvements in mystical experience intensity. Preliminary efficacy signals were encouraging, with improvements in anxiety, depression, demoralization, death anxiety, quality of life, and connectedness maintained through 24 weeks of follow-up. These findings support further investigation of retreatment protocols for partial responders, individualized dosing strategies, and the potential benefits of group-based psilocybin therapy models for psychedelic public health.

## Data Availability

The data that support the findings of this study are available on request from the corresponding author to qualified researchers but are subject to FDA Certificate of Confidentiality restrictions.
