# Supplementary material for: A phase 1 study of a second experience with Group Retreat Psilocybin Therapy for partial responders after a first experience
Source: Front Public Health. 2026 Apr 14;14:1810904. doi: 10.3389/fpubh.2026.1810904 (PMC13122983; doi:10.3389/fpubh.2026.1810904)
Supplement: Supplementary file 1 [file Data_Sheet_1.pdf]

**FRED HUTCHINSON CANCER CENTER**
**UNIVERSITY OF WASHINGTON SCHOOL OF MEDICINE**
**SEATTLE CHILDREN'S**
Current version: *v1.7 March 05, 2024*
Previous version *v1.6*

| Title of Protocol: |
|---|
| A Phase 1 Study of a Second Psilocybin Group Retreat for Partial Responders with Anxiety Associated with Metastatic Cancer |

| **Investigators List:** | | |
|---|---|---|
| **Investigator** | **Professional Title** | **Phone Number** |
| ***Anthony Back MD*** | ***Professor, University of Washington*** | [redacted] |
| ***Bonnie McGregor PhD*** | ***Founder, Orion Center for Integrative Medicine*** | [redacted] |
| **Biostatistician:** | | |
| ***Ted Gooley PhD*** | ***Professor, Fred Hutch Cancer Center*** | [redacted] |

| **IND Number:** |
|---|
| 165562 |
| **Investigationant:** |
| Psilocybin PEX010, Psilo Scientific Ltd. |
| **IND Sponsor:** |
| ***Anthony Back MD*** |

| **Supported by:** |
|---|
| Psilo Scientific Ltd is providing the investigational product. |

| **Emergency contact (24-hour) phone number:** |
|---|
| Anthony Back 206-619-4367 |

**PROTOCOL SYNOPSIS**

| | |
|---|---|
| Protocol Title | A Phase 1 Study of a Second Psilocybin Group Retreat for Partial Responders with Anxiety Associated with Metastatic Cancer |
| Protocol Numbers | *BACK004* |
| IND Sponsor (if applicable) | *Anthony Back MD* |
| Funding Sponsor (if applicable) | *Steven and Alexandra Cohen Foundation* |
| Supplier of Investigational Product | *Psilo Scientific Ltd* |
| Trial Phase | *1* |
| Trial Type | *Interventional* |
| Clinical Indication | *Cancer-related anxiety* |
| Study Objectives | 1. Test safety of a second group psilocybin retreat for participants in our now-completed study who had a partial response to their first psilocybin retreat.<br>2. Test the safety of a booster dose of psilocybin for participants who report low subjective effect at 60 min after psilocybin ingestion.<br>3. Examine efficacy of a second group psilocybin retreat on partial responders in symptoms of cancer-related anxiety and depression. |
| Study Design | *Single arm* |
| Population | *Stable metastatic cancer patients* |
| Primary Endpoints | • *To test the safety of a second group psilocybin retreat for patients with metastatic cancer who participated in BACK002, with a starting dose of psilocybin 35 mg, plus an optional booster dose of 10 mg.* |
| Secondary Endpoints | • *Symptoms of depression and anxiety using the Hospital Anxiety and Depression Scale (HADS) at 1 week, 3 weeks, 5 weeks, 8 weeks, 12 weeks, and 6 months post psilocybin-assisted group therapy.* |
| Exploratory Endpoints | • *Measurement of quality of life using the Functional Assessment of Cancer Therapy – General (FACT-G) at 1 week, 3 weeks, 5 weeks, 8 weeks, 12 weeks, and 6 months post psilocybin-assisted group therapy.*<br>• *Measurement of demoralization using the Demoralization II (DS-II) scale at 3 weeks , 5 weeks, 8 weeks, 12 weeks, and 6 months post psilocybin-assisted group therapy.*<br>• *Measurement of psychosocial functioning using the NIH_HEALS) at 3 weeks, 5 weeks, 8 weeks, 12 weeks, and 6 months post psilocybin-assisted group therapy..*<br>• *Measurement of acceptance using the Watts Connectedness Scale at 1 week, 3 weeks , 5 weeks, 8 weeks, 12 weeks, and 6 months post psilocybin-assisted group therapy.*<br>• *Measurement of retreat experience using the Mystical Experience Questionnaire, Communitas Scale, Challenging Experiences Scale, Emotional Breakthrough Scale, the Psychedelic Music Questionnaire and the Strength of Drug* |

| | |
|---|---|
| | *Effect on the day of the psilocybin.session.*<br>• *Measurement of the Group Environment Scale (Cohesion subscale), Social identification single item, Social Support scale, Purpose and Meaning Scale and Global Impression of Change scale at 1 week, 3 weeks, 5 weeks, 8 weeks, 12 weeks, and 6 months post psilocybin.* |
| Investigational Product | *Psilocybin PEX010* |
| Dose | *35 mg, with optional 10 mg booster dose* |
| Route of Administration | *Oral* |
| Regimen | *Single dose + psychotherapy group and individual sessions* |
| Treatment Groups | *This is a small group psychosocial intervention delivered at a retreat center, an extension of the same methodology used in BACK002, which demonstrated safety of the facilitator: participant ratio.* |
| Treatment Schedule/Schema | *Prior to psilocybin session there are 3 sessions of weekly group therapy and 1 session of individual therapy. Day 0 = Psilocybin session. After the psilocybin session there are 3 sessions of weekly group therapy and 1 session of individual therapy.* |
| Efficacy Assessments | *1 week, 3 weeks, 5 weeks, 8 weeks, 12 weeks, 24 weeks (6 months)* |
| Number of Subjects | *Up to 16* |
| Estimated Duration of Trial | *1.5 year* |
| Duration of Participation | *8 months* |

## Table of Contents


1.0 INTRODUCTION TO THE PROTOCOL ..... 7
1.1 Introduction ..... 7
1.2 Preclinical Data (same as BACK002) ..... 7
1.3 Clinical Data to Date ..... 8
1.4 Study Agent (same as BACK002) ..... 10
1.5 Dose Rationale ..... 11
1.6 Other Agents ..... 12
1.7 Risks/Benefits ..... 12
2.0 OVERVIEW OF CLINICAL TRIAL ..... 12
2.1 Study Objectives and Endpoints ..... 13
2.2 Study Design ..... 14
2.3 Estimated Accrual ..... 14
3.0 INCLUSION AND RECRUITMENT ..... 14
3.1 Eligibility of Women and Minorities ..... 14
3.2 Recruitment of Minority Groups ..... 14
3.3 Inclusion Across the Lifespan ..... 14
3.4 Study Population ..... 14
4.0 STUDY AGENT(S) ..... 15
4.1 Primary Investigational Agent ..... 15
5.0 SUBJECT ELIGIBLITY ..... 15
5.1 Inclusion Criteria ..... 15
5.2 Exclusion Criteria ..... 17
6.0 SUBJECT REGISTRATION ..... 18
7.0 TREATMENT PLAN ..... 18
7.2 Treatment Plan Schema ..... 18
7.11 Administration of Investigational Agent ..... 21
7.12 Concomitant Medication and Supportive Care Guidelines ..... 21
7.13 Duration of Therapy ..... 23
7.14 Duration of Follow-Up ..... 23
7.15 Dosing Delays/Dose Modifications ..... 23
8.0 SUBJECT EVALUATION ..... 23
8.1 Clinical Evaluations ..... 23
8.2 Screening and Baseline Evaluations ..... 23
8.3 Psilocybin session ..... 23
8.4 Integration visit safety checks ..... 24
8.5 Post-Treatment Follow-up Period (specify visits based on days after psilocybin session) ..... 24
8.6 Long-Term Follow-up Period (specify duration and timing) ..... 25

8.7 Plan of Action for A Participant Exhibiting Suicidality ..... 25
9.0 TOXICITY MONITORING ..... 25
10.0 SUBJECT DISCONTINUATION OF ACTIVE TREATMENT ..... 26
11.0 CONCOMITANT MEDICATIONS ..... 26
12.0 ADVERSE EVENTS ..... 27
12.1 Adverse Event ..... 27
12.2 Serious Adverse Event ..... 28
12.3 Unexpected Adverse Event ..... 29
12.4 Monitoring and Recording Adverse Events ..... 29
12.5 Grading Adverse Event Severity ..... 29
12.6 Attribution of an Adverse Event ..... 29
12.7 Adverse Event Recording Period ..... 30
12.8 Adverse Event Reporting Requirements ..... 30
13.0 CRITERIA FOR ENDPOINT EVALUATIONS ..... 31
14.0 ASSESSMENT OF EFFICACY ..... 31
14.1 Efficacy and endpoint assessment ..... 31
14.2 Method and Timing ..... 31
15.0 STATISTICAL CONSIDERATIONS ..... 31
15.1 Study Design ..... 31
15.2 Objectives and Hypotheses ..... 31
15.3 Primary/Secondary Endpoints/Hypotheses and Analytical Methods ..... 32
15.4 Randomization ..... 32
15.5 Exploratory Analysis ..... 32
16.0 DATA MANAGEMENT/CONFIDENTIALITY ..... 32
16.1 Data Type ..... 32
16.2 Related Tools, Software and/or Code: ..... 33
16.3 Standards ..... 33
16.4 Data Preservation, Access, and Associated Timelines ..... 33
16.5 Oversight of Data Management and Sharing: ..... 33
17.0 DATA AND SAFETY MONITORING PLAN ..... 34
18.0 INVESTIGATOR OBLIGATIONS ..... 34
19.0 ADMINISTRATIVE AND REGULATORY CONSIDERATIONS ..... 34
19.1 Pre-Study Documentation ..... 34
19.2 Study Site Training ..... 34
19.3 Documentation ..... 35
19.4 Access to Source Data ..... 35
19.5 Data Collection ..... 35
19.6 Protocol Interpretation and Compliance ..... 35
19.7 Study Monitoring and Data Collection ..... 35

19.8 Disclosure of Data/Publication ..... 35
19.9 Ethical Considerations ..... 36
19.10 Informed Consent ..... 36
19.11 Institutional Review Board ..... 36
19.12 Subject Privacy ..... 37
20.0 STOPPING THE STUDY ..... 37
21.0 REFERENCES ..... 39
22.0 APPENDICES ..... 41

## 1.0 INTRODUCTION TO THE PROTOCOL

### 1.1 Introduction

**Psilocybin-assisted therapy is proving to be a potent treatment for the anxiety, distress and fear of recurrence experienced by people living with cancer**. In randomized studies, psilocybin-assisted therapy conducted using an existing 2-therapist/1-patient model has shown impressive efficacy for cancer-related anxiety and existential distress.[1-3] Larger, multisite randomized trials are being launched now. In addition, psilocybin-assisted therapy is being tested for a number of other indications, including depression, substance use, obsessive-compulsive disorder, demoralization, and—in a study Dr Back is leading—symptoms of depression and burnout in doctors and nurses who developed these symptoms during their frontline work in the COVID pandemic.

**For patients with metastatic, incurable cancer, unrelieved anxiety and existential distress cause profound suffering.** Of patients with metastatic cancer, 25-50% have clinically significant anxiety.[4-6] These patients experience a sense of uncertainty, fear of future uncontrollable suffering, sense that they are burdens on the people caring for them, feelings of loneliness and isolation, and grief for the loss of their lives and opportunities missed. In longitudinal studies, existential distress and anxiety generally increases over time, and probably contribute to patients' pursuit of futile medical interventions at the very end of life.[7] Existing therapies are unsatisfactory.[8] For many of these patients, their anxiety and distress impedes their ability to participate in psychotherapy, many decline to take antidepressant medications because of the unwanted side effect of blunting positive emotions, and benzodiazepines do not seem to change the course of the anxiety.

### 1.2 Preclinical Data (same as BACK002)

**Substantial data demonstrates the safety of psilocybin (mushroom-derived, and synthesized)**

Studies in humans and nonhuman animals indicate that psilocybin has very low toxicity.[9,10] The $LD_{50}$ ranged from 285mg/kg in rats and mice.[11] The maximum tolerated dose of psilocybin in humans has not been defined or established. The best estimate of a lethal dose for pure psilocybin in humans is about 19 grams. The full dose of psilocybin that will be administered in this study is 1/100 times of the established $LD_{50}$ in nonhuman animals and in humans. A Phase I trial of psilocybin completed in 2019 at oral doses of up to 0.6 mg/kg demonstrated no serious adverse events.[12] Psilocybin is not associated with disease or damage to any organ or system.[10] More commonly, damage or disease to organs (as renal failure) is associated with mistakenly consuming poisonous mushrooms under the belief that they are psilocybin-containing mushrooms.[13]

Three cases of death possibly related to the direct toxic effects of Psilocybe mushrooms (the natural source of psilocybin) have been reported in the world literature. One fatality occurred in a post-cardiac transplant patient who ingested Psilocybe mushrooms.[14] It is not known however whether psilocybin was the proximal cause of death or whether death occurred secondary to the cardiac stimulant phenethylamine, which is also present in the mushrooms. In the French literature, another fatality linked to Psilocybe mushroom use was associated with far higher blood concentrations of the active metabolite of psilocybin than would be expected with the doses proposed in this study.[15] A final fatality from Psilocybe mushroom ingestion was mentioned in a 1961 review but was poorly characterized, with no psilocin concentrations were reported.[16]

In a review of other adverse effects of psilocybin from Psilocybe mushrooms, an additional 10 individuals were reported to have experienced accidental deaths associated with Psilocybe mushroom ingestion.[17] In most cases, these accidental deaths resulted from falls from buildings. Given the documented history of use of *Psilocybe* mushrooms in a variety of societies and cultures, that there are only 3 known fatalities at best indirectly linked to the physiologic effects of mushroom ingestion is an argument for the general safety of this natural product. Further, a recent review of

the potential harm of the ingestion of *Psilocybe* mushrooms by the Dutch medicinal advisory board found that the risk of acute or chronic toxicity was low.[17]

To date, over 2000 participants have received psilocybin under controlled conditions in scientific studies with no reports of the occurrence of a significant adverse event deemed to be associated with drug administration. The most commonly reported adverse events from the scientific literature are psychological in nature and include the induction of negative emotional states and paranoid/delusional thinking during psilocybin sessions, as well as far less frequent reports of Hallucinogen Persisting Perception Disorder (HPPD).[18] A review of studies conducted worldwide between 1999 and 2008 identified only one subject (out of 110) who experienced any persistent perceptual symptoms associated with HPPD and these symptoms were mild, brief, and resolved within three days of psilocybin exposure.[19] Rates of prolonged psychiatric symptoms of any kind following psilocybin exposure in healthy study participants are estimated to be 0.08-0.09%. As in the phase 2 studies, common physical adverse events associated with psilocybin administration include increased BP and heart rate, nausea, and headaches. In the phase 2 studies of cancer related anxiety and depression no cases of HPPD were identified and no participants developed any symptoms of paranoia or anxiety that required pharmacological interventionor anything more than reassurance from session facilitators.[1,2]

The data from uncontrolled usage are different. In a survey of 1993 individuals who engaged in unsupervised, illicit use of unknown doses of psilocybin-containing mushrooms in an uncontrolled setting, 11% put self or others at risk of physical harm. Of the respondents, 2.6% behaved in a physically aggressive or violent manner and 2.7% received medical help. Of those whose experience occurred >1 year before, 7.6% sought treatment for enduring psychological symptoms. 3 cases appeared associated with onset of enduring psychotic symptoms and 3 cases with attempted suicide.[20]

There have been no reports of physical harm in patients who have received psilocybin under controlled conditions, and no fatalities have been associated with its use in a controlled clinical trial. In addition to knowing the exact dose of medically prepared oral psilocybin consumed in the controlled clinical trial, like the proposed study is great attention is giving to the set and *setting* of the experience. The *set* refers to the emotional/cognitive/behavioral state/mindset and expectations of study participants just prior to psilocybin exposure, and the *setting* refers to the physical environment in which the exposure occurs.[21]

There are no confirmed reports of an overdose of synthesized or purified psilocybin meant for use in a clinical trial. In the United States, use of chemically synthesized psilocybin does not occur.[22] Although psilocybin in the form of mushrooms is sometimes used non-medically, medical emergencies due to psilocybin mushrooms are very rare (psilocybin is mentioned in only 0.1% of drug-related emergency department visits).[23] It can be anticipated that an overdose of psilocybin might present in a manner similar to “serotonin syndrome”. Removal of any residual gastric drug, supportive care, and cautious administration of a serotonin antagonist such as risperidone are reasonable interventions; however, to date no cases have described similar responses with respect to psilocybin, and the doses of psilocybin that might provoke these physiological effects would be many times greater than even the highest doses used in early human studies.

Since BACK002 was initiated, no new preclinical data that impact the safety of psilocybin for this population have been published.

### 1.3 Clinical Data to Date

**Psilocybin-assisted therapy can relieve anxiety and existential distress by disrupting patterns of thinking that are ruminative, catastrophic, and persistent.** The lived experience of cancer patients who have participated in psilocybin-assisted therapy studies using individual models reveal that patients feel more able to examine their fears, find meaning in their lives, and connect more deeply the people they care about.[24] While scientific studies have yet to measure medical utilization, anecdotes describe a dramatic shift in decisions away from medical

treatments that are invasive and isolating, such as mechanical ventilation in intensive care, and an increase in discussions related to advance care planning. The qualitative data, and many anecdotes published in the media, demonstrate that psilocybin-assisted therapy seems to allow many patients to hold a realistic appraisal of their prognosis while at the same time experiencing a heightened appreciation of the parts of their lives they still enjoy and cherish—and these benefits have persisted in a 4 year follow-up study.[25] While other psychotherapeutic approaches have been reported as valuable, their benefits take much longer to be experienced, typically weeks to months, and long term follow-up is sparse. Thus psilocybin-assisted therapy appears to have unique promise as a modality to treat anxiety, existential distress, and demoralization for patients with life-threatening cancer. The study proposed here addresses a population of patients with metastatic cancer whose need for relief from suffering is high.

**Previous clinical treatment models used a 2-therapist/1-patient therapy model that is extremely resource intensive, to a degree that will likely limit future access.** For example, in two highly efficacious clinical trials, 2 therapists conducted 3 preparation sessions, a medication day sessions, and 3 integration sessions with a single participant. A single study participant required at least 30 hours of direct contact. [Assuming (3 x 90 min prep sessions) + (1 x 7 hour medication session) + (3 x 90 min integration sessions] x 2 therapists]. This 2 therapist/1 patient model is likely to become a barrier to expanding access for the 2 therapist/1 patient model. Overall, a group model could ultimately provide increased access for a carefully selected and screened population.

***Note that this protocol uses the term 'facilitator' rather than therapist, but for this protocol these terms both refer to the same role on the research team.***

**Our prior study (BACK002, IND165562) was the first step towards our ultimate goal of developing a safe and efficacious group model for psilocybin-assisted therapy in a retreat setting**. The goal of the project was to test a model of 4 core facilitators (and 2 backup facilitators) for a group of participants that started at 5 and increased to 8 over the course of the study. Over 8 retreats involving 53 people, we did not have a single serious adverse event in which more than 4 participants needed 1:1 attention from the 4 core facilitators at any one time (there were 2 SAEs both unrelated to the psilocybin, one death from progressive cancer and one hospitalization for chemotherapy-related dehydration for a patient on FOLFIRINOX a week after the retreat). In preliminary analyses, this group model is also efficacious, with drops in the Hospital Anxiety and Depression Total score that are comparable to the 2 facilitator / 1 patient model.[1]

**During the group sessions in our prior study ,we observed partial responders at levels comparable to results reported by other researchers using the 2 facilitator/1 patient model.** We are defining 'partial response' as one or more of the following 4 criteria.

**Criterion 1: MEQ score that is lower than 'complete.'** About 40% of our participants did not experience a full psychedelic experience, as measured by the Mystical Experience Questionnaire. In other studies, a 60% score on their MEQ was considered a 'complete' experience, and in other studies of healthy normal, about 70% of participants were reported to have complete experiences by this definition. There are 3 potential explanations for this finding in our study are that (a) many of our participants had tapered off antidepressant medication, which leaves them less likely to have a complete experience, and (b) many were also taking active anticancer therapy; and (c) some people may need a higher dose of psilocybin.

**Criterion 2: HADS change from Prep to Day 28 that shows worsening symptoms or minimal response**. In our study , 9 participants had an *increase* (meaning their symptoms worsened) in their HADS from Prep 1 (prior to their psilocybin session) to Day 28. Also 9 participants had an improvement in the HADS Total score from Prep 1 to Day 28 that was 5 points or less. Most of these participants had complex personal issues that they addressed in their psilocybin session and did not get to the issue of their mortality.

**Criterion 3: Participants who had a HADS Total score Day 28 that was >11.** In our study, 18 participants had a Day 28 HADS Total score >11, meaning that they still had moderate or severe symptoms of anxiety and/or depression. Most of these patients had some improvement in their HADS, but our anecdotal experience was many felt like they 'still had more work to do'.

**Criterion 4**: **Participants who have experienced a recurrence in their symptoms of anxiety or depression since completing the 6 month followup** for the prior study. The study team has received 2 queries from prior participants (who did not know a second experience study was planned), asking for advice about how to manage recurrent anxiety.

There has been no research that addresses these 'partial responders' published in the scientific literature.

**In our prior study, we also observed anecdotally that tapering antidepressants may have caused harm.** There are two types of potential harm. First, most of the participants who tapered off their antidepressants in our study reported during their weekly check-ins that they were feeling 'more edgy' or 'a little worse' or 'more cranky.' This observation is consistent with a large body of data describing increased symptomatology for patients tapering antidepressants. The second potential harm is that these increased symptoms may have created a barrier within their own minds impeding the psilocybin effect—if a participant is trying to control their anxious thoughts, they are less likely to allow the drug effect to occur and what we observe is that they have difficulty 'dropping in' to the experience.

**The prior use of antidepressants, even if tapered, may attenuate the treatment effect of psilocybin.** The existing data from clinical trials to address this possibility is scant and conflicting. One secondary analysis of a clinical trial comparing psilocybin-assisted therapy to escitalopram that suggests that prior antidepressant use, tapered off for the study, attenuates the psilocybin effect.[26] Another small clinical trial that did not require antidepressant tapering did not identify concurrent antidepressant use as factor associated with a lower treatment effect and noted no additional safety issues.[27] A retrospective observational study and a prospective naturalistic study, with much larger sample sizes but unverified doses of psilocybin, indicates that concurrent antidepressant use attenuates the treatment effect.[28] In our prior study (BACK002), we observed a reduced MEQ score and HADS outcome score in participants who had been tapered off an antidepressant prior to study participation.

**Note that the participants we plan to recruit from our prior study are a useful sample to investigate the effect of a second experience and also the effect of not requiring an antidepressant taper.** These participants were required to have no pre-cancer history of anxiety or depression treatment with medications; had a first experience without SAEs, and if on an antidepressant at study entry were only on one medication at moderate dose, often for less than 3 years. (In contrast, patients with treatment-resistant depression in studies of psilocybin-assisted therapy often are on multiple meds for 10 years or more.) Also, since these participants will all be observed for 24 hours after the ingestion of their initial dose, we will have ample opportunity to identify and characterize any adverse events.

**There is a gap in knowledge about whether the benefits of psilocybin-assisted therapy could be maximized by increasing the 'dose' of therapy from one episode to two episodes of psilocybin-assisted therapy. This increased dose might be able to obviate the need for tapering antidepressants.** For the current study, in order to address pre-existing factors that may have contributed to a partial response, we propose two modifications to the dose of psilocybin we used in our prior study:

1) The starting dose in this study will be higher, 35 mg psilocybin (compared to 25 mg in the prior study).
2) After the initial 35 mg dose, for participants who are not feeling a drug effect at 60 min, we will offer a extra safety screening that if passed will make available a booster dose of 10 mg psilocybin.

The rationale for these higher doses is below in Section 1.5, Dose Rationale.

## 1.4 Study Agent (same as BACK002)

**Psilocybin is a naturally occurring compound**, found in a variety of mushroom species collectively called 'magic mushrooms' in lay publications. According to the most recent and comprehensive review, serotonergic psychedelic medications "are generally considered to be physiologically safe molecules" that chiefly alterconsciousness.[29] Recent reviews and searches of the literature indicate that psilocybin – either in the form

of mushrooms, or synthesized psilocybin -- is not associated with harm or damage to any organ or system in the body.[18] Psilocybin can produce changes in blood pressure and heart rate, but these changes are not as strong or consistent as those seen after psychostimulants (amphetamines), and sometimes these changes only occur at one time point.[29] More consistently, psilocybin can produce rapid and intense changes in mood including periods of anxiety or panic. Both these physiological and psychological effects are transient and do not last beyond the duration of drug effects.

**The psilocybin that will be used in this study, PYEX psilocybin, is a partially purified fraction of the extract of Psilocybe cubensis mushroom fruiting bodies** that is produced by Psilo Scientific Ltd. (Vancouver BC), in a patented process that yields a pharmaceutical grade botanical drug that meets FDA guidance for a botanical drug suitable for a clinical investigation. The drug product to be used is PEX010, 25 mg psilocybin capsules, and it is manufactured under cGMP conditions. Further CMC information is available in the accompanying Investigator's Brochure describing PEX010. In a variety of studies, researchers leading clinical trials using pharmaceutical grade psilocybin at this dose took precautions similar to the ones we will use in this study, and observed no reactions requiring pharmacological intervention.[2,30] This preparation of psilocybin is being used in over 25 studies in North America, Europe, and Australia, including an ongoing study at the University of California San Francisco (personal communication, Anderson B., May 2024).

## 1.5 Dose Rationale

**The starting dose of psilocybin to be used in this study will be 35 mg psilocybin** PEX010. In our prior study we used 25 mg psilocybin because of a review of experience at the Johns Hopkins Center for Consciousness Studies.[31] However that study produced partial responders also, and no further investigation regarding partial responders has been conducted. We note that in our review of the prior studies for patients with cancer, prior studies did use a slightly higher dose of 30 mg (rather than 25 mg as in our prior study), and that clinical trials of other conditions (notably substance use disorder) have doses as high as 40 mg of psilocybin.[32,33] In addition, the maximum dose allowed in Oregon, under that state's regulated use program, is up to 50 mg. Other clinical trials have demonstrated the safety of psilocybin up to 60 mg. From a recent review: "Several dose-escalating studies have tested the subjective psychedelic effects in supratherapeutic doses, e.g., 50–60 mg or 5–6 grams, and found positive results with little-to-no safety concerns."[34-36]

**We also propose a booster dose of 10 mg be available after safety screening** from 60-90 after the first dose. The booster dose will be available to any participant in this study, regardless of concurrent antidepressant use, because we hypothesize that a higher dose may be required for some people who are not on antidepressants, and because a higher dose may overcome the attenuation effect of concurrent antidepressants. Note that in the retrospective observational study, participants reported that the reduced treatment effect of antidepressant use could be overcome with a larger dose of psilocybin in many cases.[28]

The scientific justification for the higher starting dose and additional potential booster dose is grounded in empirical studies. First, **the participants in this study are able to make a subjective determination of whether their response is partial** because they have already had a personal experience with 25 mg psilocybin that they compared with 4-7 peer participants at the last retreat. Second, **a partial subjective response is associated with less improvement** in prior psilocybin studies have established that on symptoms of depression or anxiety (Ross, 2016). Third, t**he subjective response to psilocybin is dose-dependent** in a dose-finding study of psilocybin (Studerus, 2011). Thus, if a participant judges that they are having a partial response, it is likely that they will experience less benefit—so if safety can be maintained, it is reasonable to consider increasing their psilocybin dose.

The timing of considering a booster dose at 60-90 min is based on pharmacokinetic studies that measured subjective effect at predefined timepoints (Holze, 2023; Carbonaro, 2017). These studies demonstrated that the subjective effect of psilocybin rises rapidly after oral administration so that at 60 minutes about 60% of participants are experiencing a drug effect, with 80% reporting a drug effect at 2 hours (120 min). At the individual participant level, **participants who have not reported a subjective effect at 60 min appear much more likely to**

**have a lower intensity peak experience**. Thus, these studies suggest that 60 minutes is a reasonable time to begin consideration of a booster.

**To be eligible for a booster dose, we will require a safety screen** immediately before the booster dose is offered. We will require that to be eligible for a booster dose that a participant report that they are having little to no subjective effect, that they are able to walk up to a PI evaluation station in the room where the dosing session is held, and be evaluated by the PI for hypertension, hyperthermia, and other evidence of serotonin syndrome (hyperreflexia, clonus), or any other medical issue that might be a contraindication for the booster dose (including nausea, dissociation, derealization). A new ECG onsite will be required, and the QTc interval must be <450 ms. Study participants who receive a booster dose will receive a total psilocybin dose of 45 mg. Specific criteria for the booster dose are listed below in Section 8.3.

### 1.6 Other Agents

There are no other pharmacologic agents used in this study. The study intervention includes psychotherapy sessions that are similar to established small group psychosocial interventions for cancer patients.[1,37,38]

### 1.7 Risks/Benefits

**Expected benefits for participants** include: reductions in symptoms of anxiety, depression, demoralization, existential anxiety; increases in feelings of well-being and spiritual connectedness. Possible benefits for society include establishment of safe procedures for psilocybin-assisted therapy in groups, and the advancement of scientific knowledge in the area of psychedelic-assisted therapy.

**The expected benefits outweigh the risks** for study participants because: symptoms of depression, anxiety, demoralization, existential anxiety can lead to severe suffering for patients with metastatic cancer, and the existing treatment options have significant limitations. For example, about 30% of cancer patients with depressive symptoms respond to conventional antidepressant therapy. For patients with demoralization, antidepressants have no known efficacy. For patient with metastatic cancer, conventional psychotherapy has modest effects, yet requires a substantial investment of time and energy which can be difficult for patients with metastatic disease. While the evidence for psilocybin-assisted therapy is limited, existing randomized studies using an individual patient approach show responses that are larger in magnitude, more rapid, and more prolonged than existing antidepressant or psychotherapy alone studies. Thus this study, which attempts to make psilocybin-assisted therapy more accessible, and is designed with multiple safety features, seems reasonable to propose to patients with metastatic cancer.

## 2.0 OVERVIEW OF CLINICAL TRIAL

This is a phase 1 study of a *second* episode of psilocybin-assisted group therapy for cancer-related anxiety and distress in patients with metastatic cancer that will measure safety, feasibility, and efficacy in order to establish evidence-based parameters for a larger study of this group model of psilocybin-assisted therapy.

Compared to BACK002, which was the study where participants had their first episode of psilocybin-assisted therapy, the differences are:

--eligibility criteria include participation in BACK002 and the criteria for partial responders described above

--a starting dose of psilocybin 35 mg and a booster dose of 10 mg if a safety assessment is ok

--4 post-dose integration meetings (rather than the 3 integration meetings in BACK002) at 2 week intervals to enable meetings and outcomes measures to be scheduled on the same day to reduce participant burden from contacts from study staff.

## 2.1 Study Objectives and Endpoints

2.1.1 **Primary Objective.** To test the safety of a second group psilocybin retreat for patients with metastatic cancer who participated in BACK002, with a starting dose of psilocybin 35 mg, plus an optional booster dose of 10 mg.

2.1.1.1 **Primary Endpoint.** Adverse events will be measured using validated instruments for side effects using the Common Terminology Criteria for Adverse Events (CTCAE) commonly used in cancer clinical trials.

2.1.2 **Secondary Objective.** To explore the efficacy of the group model of psilocybin-assisted therapy on symptoms of anxiety and depression at 1 week, 4 weeks, 12 weeks,

2.1.2.1 **Secondary Endpoint.** The primary measurement of anxiety and depression will be Hospital Anxiety and Depression Scale at 1 week, 4 weeks, 8 weeks, 12 weeks, and 24 weeks.

2.1.3 **Exploratory Objective 1.** To explore the efficacy of the group model of psilocybin-assisted therapy on symptoms of demoralization.

2.1.3.1 **Exploratory Endpoint 1. Demoralization will be measured using the DS-II scale at** 1 week, 4 weeks, 8 weeks, 12 weeks, and 24 weeks.

2.1.4 **Exploratory Objective 2.** To explore the efficacy of the group model of psilocybin-assisted therapy on symptoms of quality of life.

2.1.4.1 **Exploratory Endpoint 2. Quality of life will be measured using the FACT-G and Adjustment Disorder scales at** 1 week, 4 weeks, 8 weeks, 12 weeks, and 24 weeks.

2.1.5 **Exploratory Objective 3.** To explore the efficacy of the group model of psilocybin-assisted therapy on psychosocial functioning and connectedness.

2.1.5.1 **Exploratory Endpoint 3. Cancer-related post-traumatic growth will be measured using the NIH-HEALS scale and the Watts Connectedness Scale at** 4 weeks, 8 weeks, 12 weeks, and 24 weeks.

2.1.6 **Exploratory Objective 4.** To explore the efficacy of the group model of psilocybin-assisted therapy on death anxiety.

2.1.6.1 **Exploratory Endpoint 4. Death anxiety will be measured using the Death and Dying Distress Scale at** 4 weeks, 12 weeks, and 24 weeks.

2.1.7 **Exploratory Objective 5.** To explore the quality of the group experience of the group model of psilocybin-assisted therapy.

2.1.7.1 **Exploratory Endpoint 5. The quality of the group experience will be measured using the Mystical Experience Questionnaire, Emotional Breakthrough Inventory, Communitas Scale, and Challenging Experiences Questionnaire, and the Psychedelic Music Questionnaire on the day of the psilocybin session only.**

2.1.8. **Exploratory Objective 6**. To pilot process measures that describe aspects of social identity that may analyzed for correlation in future, larger studies.

2.1.8.1. **Exploratory Process Measures** will include: the Strength of Drug Effect (2 item VAS), Group Environment Scale—Cohesion subscale (9 items), Social identification Scale (1 item), Social Support (8 items), Purpose and Meaning scale (5 items), and Global Impression of Change (1 item).

### 2.2 Study Design

This is a single arm, intervention study open only to participants who had a partial response (defined earlier) in the prior BACK002 study, in which participants were given a first episode of psilocybin-assisted group therapy. The present study is designed to offer partial responders a second episode of psilocybin-assisted group therapy with a higher dose of psilocybin and a possible booster dose to improve “complete experience” rates.

### 2.3 Estimated Accrual

This study will accrue up to 16 participants over 2 retreats. Note that our prior study BACK002 established that 8 participants / 4 core facilitators + 2 backup facilitators is a participant/facilitator ratio that resulted in zero instances when psilocybin-related distress during the psilocybin session could not be addressed by the 4 core facilitators.

## 3.0 INCLUSION AND RECRUITMENT

### 3.1 Eligibility of Women and Minorities

Individuals of any sex, gender, race, or ethnicity are eligible for this study.

### 3.2 Recruitment of Minority Groups

The study recruitment strategy will reflect the enrollment of BACK002, which did include a diverse group of individuals.

### 3.3 Inclusion Across the Lifespan

In BACK002, the age range was 26-81.

### 3.4 Study Population

Existing studies indicate that older age is associated with decreasing anxiety for patients with metastatic cancer, but the risk of depression does not vary with age.

A 2021 study of Central Puget Sound demographics showed that 37% are people of color, and 63% are White non-Hispanic/Latino. Among people of color: Asians (14%); Hispanic (10%); Black (6%); American Indian/Alaska Native (1%); Native Hawaiian or Other Pacific Islander (0.8%)

Projected Target Accrual
ETHNIC AND GENDER DISTRIBUTION CHART

| TARGETED / PLANNED ENROLLMENT: Number of Subjects | | | |
|---|---|---|---|
| Ethnic Category | Sex / Gender | | |
| | Females | Males | Total |
| Hispanic or Latino | 1 | 0 | 1 |
| Not Hispanic or Latino | 7 | 7 | 15 |
| Ethnic Category Total of All Subjects* | 8 | 8 | 16 |
| Racial Categories | | | |
| American Indian / Alaska Native | 0 | 0 | 0 |
| Asian | 2 | 1 | 3 |

| | | | |
|---|---|---|---|
| Native Hawaiian or Other Pacific Islander | 0 | 0 | 0 |
| Black or African American | 1 | 0 | 1 |
| White | 5 | 7 | 12 |
| More Than One Race | 0 | 0 | 0 |
| Racial Categories: Total of All Subjects* | 8 | 8 | 16 |

## 4.0 STUDY AGENT(S)

### 4.1 Primary Investigational Agent

Psilocybin, is a 4-hydroxy-N,N-dimethyltryptamine and occurs in nature in many species of mushrooms, including the genera Psilocybe, Conocybe, Gymnopilus, Panaeolus, and Strophparia. Its chemical formula is $C_{12}H_{17}N_2O_4P$. Psilocybin is a potent agonist at 5-HT2A/C, and their binding potency to these receptors correlates with human potency as hallucinogens [7]. Psilocybin has been administered to normal volunteers to measure physiological and psychological parameters at doses ranging from 0.045mg/kg to0.43mg/kg [45; 51]. It has also been safely administered to patients with OCD at doses ranging from 0.025mg/kg to 0.3mg/kg.

Psilocybin PEX010 is a naturally occurring compound that after oral administration is metabolized to psilocin, which reacts antagonistically with serotonin type 2A (5-HT2A) receptors to produce the psychological and spiritual experiences that patients often report. Psilocybin PEX101 is a partially purified fraction of the extract of Psilocybe cubensis mushroom fruiting bodies that is produced by Psilo Scientific Ltd. (Vancouver BC), in a patented process that yields a pharmaceutical grade botanical drug that meets FDA guidance for a botanical drug suitable for a clinical investigation. PEX010 will be provided to Dr. Back for this study in 10 mg and 5 mg psilocybin capsules that have been manufactured under cGMP conditions.

4.1.1 The investigational agent will be shipped directly to Harborview Investigational Drug Service to Dr. Back's attention and will remain unopened in the narcotics cabinet for accountability by Dr. Back. Harborview IDS is working with Dr. Back on his current psilocybin studies in the same capacity.

4.1.2 The investigational agent will be stored at room temperature at the Harborview Medical Center IDS, in a locked cabinet in the narcotics vault that has been inspected and approved for storage of psilocybin by the DEA and the Washington State Department of Health.

## 5.0 SUBJECT ELIGIBLITY

### 5.1 Inclusion Criteria

5.1.1 Participation in BACK002, with outcome measures that showed: a low MEQ score, OR a small (or negative) HADS change score, a last HADS score that was 11 or greater, OR who have experienced a recurrence in their symptoms of anxiety or depression since completing the 6 month follow-up.

5.1.2 A diagnosis of metastatic solid tumor, or incurable hematologic malignancy that has been accepted by a physician in a medical record.

5.1.3 Measurable disease is not required.

5.1.4 Previous treatment with chemotherapy: There are no minimum or maximum prior lines of chemotherapy.

5.1.5 18-85 years of age.

5.1.6 Required performance status, including the appropriate scale. ECOG 0-2.

5.1.7 Acceptable organ and marrow function includes:
Hematocrit >20, Plt >20K, Liver function tests 1.5x normal, Creatinine 1.5x normal.

5.1.8 Subjects of childbearing potential must be willing to use an effective contraceptive method from study enrollment until at least 1 month after receiving the investigational agent(s).

5.1.9 Must be at least 4 weeks after surgery or radiotherapy at study entry, but can be receiving oral or iv chemotherapy if those schedules can be adjusted around the medication session date;

5.1.10 Motivated to participate in a group study and able in the research team's judgment to participate in the small group effectively;

5.1.11 On pre-enrollment screening tests, they will have clinically significant anxiety or depressive symptoms as defined by a score of 11 or greater on the HADS-Total.

5.1.12 English speaking – able to understand the process of consent and the risk andbenefits associated with the study, and able to give written informed consent. This is a pilot study, and if future larger studies are designed, consideration will be given for non-english-speaking subjects.

5.1.13 Must be willing to sign a medical release for the investigators to communicate directly with their treating clinicians (mental health professional or oncologist) and doctors to confirm a medication and/or medical history.

5.1.14 Must provide at least one adult who is in contact with the participant at least once a day when the participant is at home who is able to verbally monitor participant-reported changes in the behavior and able to notify research staff of behavior changes that may require research staff assessment.

5.1.15 (In BACK002, participants were required to taper off SSRIs in this study they will be allowed to continue.) Must provide a review of any SSRI use since completing BACK002.

5.1.16 Must avoid taking any psychiatric medications or starting a new psychiatric medication during the study. Should participant's doctor recommend starting a new psychiatric medication, participant will be required to notify the study team and the subject would withdraw from the study. (Use of prn benzodiazepines is allowed but high dose chronic benzodiazepine use must be reviewed by the PI. Use of prn gabapentoids is allowed but high dose chronic gabapentoid use must be reviewed by the PI.)

5.1.17 Must provide a contact (relative, spouse, close friend, or other caregiver; can be the same person as in 5.1.13) who is willing and able to be reached by the research team in the event that the participant becomes suicidal.

5.1.18 If the potential participant is of childbearing potential, they must have a negative pregnancy test at baseline and prior to the medication dosing session, and must agree to use adequate birth control.

5.1.19 Are willing to commit to preparation sessions, medication dosing sessions, integration sessions, to complete evaluation instruments and commit to be contacted for all necessary telephone contacts.

5.1.20 Must have had serum lab tests within 2 weeks of the retreat showing values for potassium (K), magnesium (Mg), and Calcium in the normal range. (Electrolyte repletion and rechecking of serum labs is allowed to establish eligibility.)

## 5.2 Exclusion Criteria

5.2.1 Brain metastases that have not been treated.

5.2.2 Uncontrolled or concurrent illness including, but not limited to, ongoing or active infection, symptomatic congestive heart failure, unstable angina pectoris, cardiac arrhythmia, or psychiatric illness/social situations that would limit compliance with study requirements.

5.2.3 Pregnancy, breastfeeding, or expecting to conceive or father children for the duration of the trial through 30 days after receipt of investigational agent(s).

5.2.4 Personal or immediate family history of schizophrenia, bipolar affective disorder, delusion disorder, paranoid disorder, or schizoaffective disorder;

5.2.5 Suicidal ideation with a C-SSRS $\geq$ 3;

5.2.6 Current substance abuse disorder (although prospective subjects will not be excluded for reasonable alcohol use that does not meet criteria for alcohol use disorder or marijuana use that does not meet criteria for substance use disorder);

5.2.7 Unstable neurological or medical condition; history of seizure, chronic/severe headaches;

5.2.8 Any use of psychedelic drugs in high doses (psilocybin >2 grams of dried mushrooms, LSD >200 micrograms) within the prior 3 months (microdosing will not require exclusion but participants would have to agree to discontinue microdosing 1 month before study entry);

5.2.9 Use of tramadol, due to the potential for serotonin syndrome with concomitant use of psilocybin.

5.2.10 Individuals who are on MOAI (monoamine oxidase inhibitors) or who have a known sensitivity to the drug or its metabolites. Psilocybin is contraindicated in medications that are known UGT (UDP-glucuronosyltransferase) enzyme modulators.

5.2.11 Baseline prolongation of QT/QTc interval (e.g., demonstration on an eligibility 12-lead ECG of a QTc interval >450 milliseconds (ms).

5.2.12 A history of additional risk factors for Torsade de Points (including but not limited to: heart failure, hypokalemia, family history of Long QT Syndrome) .

5.2.13 The use of concomitant medications that prolong the QT/QTc interval.

5.2.14 Any history of cardiovascular disease such as history of myocardial infarction or congestive heart failure or cardiac arrhythmia.

5.2.15 The use of concomitant medications that prolong the QT/QTc interval.

5.2.16 Concomitant use of efavirenz (an antiviral) which cannot be tapered.

5.2.17 Concomitant use of serotonin-acting supplements due to their potential for interaction with psilocybin, including 5-HTP, St John's Wort, and 'brain food' supplements.

## 6.0 SUBJECT REGISTRATION

Subjects will be registered by the Fred Hutch/UW Study Coordinator and entered into the Clinical Trial Management System (CTMS, OnCore). A complete, signed study consent and HIPAA authorization are required for registration.

Subject IDs will be assigned by CTMS data entry.

Signed ICFs must remain in each subject's chart and must be available for verification by monitors or regulatory agencies at any time.

## 7.0 TREATMENT PLAN

**7.1 Overview.** This study is an extension of our prior study BACK002 which defined safety parameters for the group model facilitator-to-participant ratio. In the current study, we will examine safety of 3 other parameters of a group psilocybin model, including a second experience, a higher dose of psilocybin with an optional booster dose, and the absence of a requirement to taper antidepressant medications started since finishing BACK002.

### 7.2 Treatment Plan Schema

The general treatment schema is similar to BACK002. The differences will be in the eligibility determination, and dose of psilocybin during the psilocybin session, and the timing of the group integration visits, which are spaced every 2 weeks to coincide with questionnaire administration. In this study, the group integration visits will total 4, and will occur on Day 1, Week 1, Week 3, Week 5, which is one more integration visit than in the prior study. (In BACK002, the group integration visits totaled 3, on Day 1, Week 1, and Week 2.)

**SCHEMA**

Eligibility determination

↓

REGISTRATION

↓

3 Group Preparation Therapy visits
1 Individual Preparation Therapy visit

↓

Psilocybin Session

↓

1 Individual Integration Therapy visit
4 Group Integration Therapy visits

*(end of study treatments)*

| ***Outline of Treatment Schedule*** | |
|---|---|
| ***Day*** | ***Treatment*** |
| *-14 (+/-  4 days)* | *Group Preparation First session (video)* |
| *-7 (+/- 4 days)* | *Group Preparation Second session (video)* |
| *-1 day* | *Group Preparation Third session (in person)* |
| *-1 day* | *Individual Prep Visit (in person)* |
| *0* | *Psilocybin Session* |
| *1 day* | *Group Integration First session (in person)* |
| *1 day* | *Individual Integration Visit (in person)* |
| *8 (+/- 4 days)* | *Group Integration Second session (video)* |
| *22 (+/- 4 days)* | *Group Integration Third session (video)* |
| *36 (+/- 4 days)* | *Group Integration Fourth session (video)* |

**7.3 Rationale. This group intervention structure contains all the safety features of BACK002, and includes one additional Integration session.** These safety features include: thorough screening at the time of enrollment; patient education regarding expectations, individual prep and integration session in addition to the group sessions. What has changed from BACK002 to this study (BACK004), is the timing of the integration sessions. In BACK002, the last integration session was 2 weeks after the psilocybin session. In this study (BACK004), the last integration session is 5 weeks after the psilocybin session. This change is in response to our observation that participants required support for a longer duration after the psilocybin session than BACK002 provided.

**7.4 Group size considerations.** Based on our findings in BACK002, we designed the current study to have group sizes of 8 (or up to 8) participants, with 4 core facilitators and 2 backup facilitators. Because we are increasing the dose of psilocybin in this study we will retain the same number of backup facilitators as in BACK002.

**7.5 Rationale for facilitation team. The 4-person facilitation team is a clinically practical, adaptable model that could set a standard for future care and has been empirically established as safe in BACK002**. This team includes two lead facilitators and two associate facilitators, and their roles will be the same as in BACK002.

--**Lead facilitator A** will (1) keep an eye on the room and allocate facilitator resources to participants who could benefit from attention, and (2) will guide participants with complex or intense emotional reactions to psilocybin. This role will be filled by the PI Dr Back for most of the groups. The lead facilitators (A and B) will be licensed healthcare providers with graduate-level professional training and clinical experience in psychotherapy. The lead facilitators (A or B) must be licensed to practice independently. Examples of acceptable professional credentials include clinical or counseling psychologist (PhD or PsyD), psychiatrist or other physician (MD or DO), Masters of Social work (MSW), licensed marriage and family therapist (LMFT), Masters Licensed Clinical Professional Counselor (LCPC), or licensed Psychiatric Nurse Practitioner (Psychiatric NP).

--**Lead facilitator B** will have the same responsibilities with the priorities reversed. The lead facilitators will work together to lead the team.

--**Associate facilitator C** responsibilities will focus on guiding participants with emotional reactions that are less complex or intense. The associate facilitators must have a bachelor's degree and at least 1 year of clinical experience in a licensed mental health care setting. In addition, a licensed physician be present on-site in the event of a physiological or psychiatric emergency.

--**Associate facilitator D** responsibilities will share guiding responsibilities with A, and also provide logistical support for trips to the bathroom, water, blankets, and ways of providing comfort.

***Note that this protocol uses the term 'facilitator' rather than therapist, but for this protocol these terms both refer to the same role on the research team.***

**7.6 The dose of psilocybin will be increased to address the partial drug effect of the psilocybin 25 mg dose used in BACK002.** Extensive review of the prior studies examining the use of psilocybin-assisted therapy for cancer reveals that most studies actually used an equivalent of 30 mg psilocybin, slightly higher than the 25 mg used in BACK002, and that these studies all had a proportion of partial responders in the range of 30%. No studies have addressed a second psilocybin-assisted therapy experience.

The rationale for starting the current study (BACK004) with 35 mg is because: 1) our clinical observation from BACK002 is that many patients with partial responses appeared to have an insufficient drug effect during their psilocybin session; and 2) the safety of psilocybin as an oral agent has been well established for doses well over 35 mg. (ref)

**7.8. A 'booster' psilocybin dose will be available from 60-90 min after the initial dose, after a safety check.** Since it is unknown whether the planned starting dose of 35 mg psilocybin will be adequate for partial responders, especially those who are on antidepressants, we will offer a booster dose of 10 mg psilocybin starting 60 min after ingestion of the initial dose, up until 90 min. After 90 min, the booster dose will no longer be available as this may lengthen the participant's time under the influence of psilocybin to longer than is practical in our group setting. The booster dose will be based on Facilitator A's clinical judgement and the participant's report of incomplete drug effect, as there is no reasonable questionnaire or other assessment available.

The rationale for the booster dose of 10 mg is as follows: in anecdotal settings, booster doses are commonly used in doses that range from (an equivalent) of 7 mg to 20 mg. There are no clinical trial data available from these settings, although naturalistic studies document the safety of these booster doses. However, in clinical trials recently, a total dose of 40 mg psilocybin has been used with excellent safety, and in Oregon, where psilocybin can be used legally with restrictions, the upper dose limit is 50 mg. In this study, a patient who

receives a booster dose would receive psilocybin 35 mg + 10 mg = 45 mg, which falls in the range of current use in clinical trials and in Oregon. (Additional scientific rationale is in Section 1.5, page 11)

**Prior to administering a booster dose, a clinical safety check will be performed** to rule out hypertension or serotonin syndrome or prolonged QTc, and will include:

--blood pressure,
--temperature,
--physical exam for severe diaphoresis, hyperreflexia, and clonus.[39] The Hunter criteria for serotonin syndrome will be used.
--12-lead ECG with QTc measurement

**The decision-making criteria for offering the booster dose will be as follows:**

- If hypertension with BP >150 mm Hg systolic or >90 mm Hg diastolic, a booster dose will *not* be offered.
- If clinical suspicion of serotonin syndrome using the Hunter criteria is detected, a booster dose will *not* be offered. (Hunter criteria include the presence of one of the following classical features or groups of features: spontaneous clonus; inducible clonus with agitation or diaphoresis; ocular clonus with agitation or diaphoresis; tremor and hyperreflexia; or hypertonia, temperature above 100.4° F (38° C), and ocular or inducible clonus.)
- If the QTc is >450 ms, a booster dose will *not* be offered.
- If a participant has a low subjective sense of experiencing a psilocybin effect AND passes the safety check, a booster dose will be offered with water.

**After a booster dose is administered, additional monitoring will be performed:**

- BP will be checked 2 hours after the booster dose. If BP is >150 systolic or >90 diastolic, BP will be checked hourly until it returns to baseline, and then every 2 hours until 6 hours after psilocybin administration.
- 12-lead ECG will be checked 2 hours after the booster dose. If the QTc >450, an ECG will be checked at 4 hours, 6 hours, and 8 hours until the QTc has returned to baseline. (This timetable is based on data from QT prolongation after psilocybin in Dahmane et al, Clinical Pharmacology in Drug Development 2021, 10(1) 78-85, 2021.) If the QTc is decreasing but is not back to baseline at 8 hours, the ECG will be rechecked before the participant goes to bed and again in the morning. If the QTc is >450 ms and *not* decreasing by bedtime, the QTc will be rechecked at 4 hour intervals during the night. The QTcF will be used as it is more reliable than the QTcB.(Dahmane, 2021)
- No participant will receive more than psilocybin 45 mg total.
- The portable ECG machine used has disposable electrodes that can be left on the participant's body during the psilocybin session so that a 12-lead ECG can be done immediately for any symptom of palpitations, near-syncope, or syncope or any investigator suspicion.

### 7.9 Administration of Investigational Agent

The investigational agent in this study, Psilocybin PEX010, will be administered during the psilocybin session, orally, in capsules, with lukewarm lemon ginger tea.

#### 7.9.1 Concomitant Medication and Supportive Care Guidelines

***Adverse event management during the psilocybin session***. Reviews of the published literature on synthesized and botanical psilocybin indicate that the physiological and psychological effects are transient. The patient will be monitored at all times by trained research and/or medical staff. The study facilitator team will remain in attendance during the entire session. On the medication day, participants will be reminded that they have agreed to remain supervised by the study facilitators until the acute effects of psilocybin have subsided, and all participants will be

accompanied by a facilitator to dinner after the medication session, and then to their individual rooms for bedtime. There will be a study staff person available through the night for any concerns that a participant might raise after the medication session.

Most study procedures can and will be immediately stopped if participants request or if they are exhibiting significant distress. However during the psilocybin session, once participants have taken the psilocybin, the psychological effects cannot be immediately terminated and must be managed until the psilocybin effect has worn off. The medication day session with psilocybin will be performed in a non-hospital setting that minimizes distraction and interruption, with a team of specially trained study facilitators and backup facilitators, and the participant will be monitored by specially trained facilitators who will have the skills to provide reassurance and a safe environment for the duration of the session.

If during the medication session, a participant does experience extreme distress, the PI may prescribe a designated 'rescue' medication in the event of symptoms that require it during or after the medication dosing session. Rescue medications may be a benzodiazepine, antihypertensive, or antinausea medication, according to the physician's clinical judgment.

Following the Investigator's Brochure, although there have been no reports of their use in well-reported clinical trials with oral psilocybin, medications will be available for the treatment of causal symptomatic hypertension, agitation, or severe psychosis.

If any participant develops an adverse reaction to the psilocybin as reported by them or as assessed by a member of the research team, we will ensure that the participant receives appropriate medical or psychiatric care. The level and exact type of care will depend on whether the adverse event occurs during the medication session where study facilitators will be in attendance or after the medication session when the participant is at home. Finally, the participant may be withdrawn from the study if his/her withdrawal is in the best interests of either the participant or of the research (e.g., due to a medical condition making the procedures unsafe or any condition making interpretation of the results difficult).

If a participant who receives a booster dose develops asymptomatic QTc prolongation >450 ms, they will receive extra monitoring as described in Section 7.8 above. If QTc prolongation lasts longer than 6 hours, an on-call cardiologist will review the ECGs and clinical condition of the participant with the PI. As noted in Section 7.8, the QTcF will be used.

If a participant who receives a booster dose develops symptoms that could be related to an arrhythmia, such as palpitations, near-syncope, or syncope, they will have a 12-lead ECG immediately, and the on-call study cardiologist will review the ECG and clinical condition of the participant with the PI immediately.

If a participant's ECG shows evidence of a ventricular arrhythmia, such as premature ventricular contractions or any ventricular arrhythmia, the on-call cardiologist (a University of Washington cardiologist working for this study) will review the ECG and clinical condition of the participant with the PI to determine whether ACLS transport to the nearby Emergency Room is indicated.

***Overnight monitoring for safety if an adverse event is occurring***: The PI and study facilitators will closely monitor the participant's medical status during all study interventions, and have the ability to monitor the patient overnight. All participants will be participating in this study at a rustic retreat center where an overnight stay after the psilocybin session is required. A night call clinician is always available in-person throughout the night to address any participant concerns. No published psilocybin study done since 2015 has reported the need for overnight hospital admission after a medication session involving psilocybin and while an overnight hospitalization will be available in this study, and the PI has not heard of any anecdotal cases of overnight observation being required in a clinical trial setting.

***Emotional Responses and Discomfort During the Psilocybin Medication Session, or During Preparation or Integration Sessions***: The study facilitators will be specially trained to work with cancer patients and also in working with group psilocybin experiences. The facilitator manual outlines first- and second-line interventions for emotional responses or any psychological reactions that may be experienced. In studies since 2000 conducted at academic research centers, no participants required 'rescue' medication for psychological reactions during the medication session or in preparation or integration sessions—emotional distress did occur, but was successfully managed with non-pharmacologic psychotherapeutic interventions.

***Inappropriate Interactions with Study Facilitators.*** At all times during in-person preparation, medication day, and integration sessions, a therapist will not be alone and unmonitored with a participant during the psilocybin session at any time.

### 7.10 Duration of Therapy

Participants in this study will receive one psilocybin session, along with associated therapy sessions. The entire duration of this psilocybin-assisted therapy from Prep 1 through Integration 4 is approximately 8 weeks.

### 7.11 Duration of Follow-Up

Participants will receive questionnaires to complete at 1 week, 3 weeks, 5 weeks, 8 weeks, 12 weeks, and 24 weeks after their psilocybin session.

### 7.12 Dosing Delays/Dose Modifications

Dosing delays are not applicable to this study as the psilocybin session occurs only once during this study.

## 8.0 SUBJECT EVALUATION

### 8.1 Clinical Evaluations

Clinical evaluations take place according to the Study Calendar (Appendix A).

### 8.2 Screening and Baseline Evaluations

Screening/Baseline Visits will occur within 2 months of the first zoom prep session. Procedures to be performed during the Screening/Baseline Visit are as follows:

- Physical Examination
- Neurological Exam
- Blood pressure
- Performance Status (ECOG)
- Labs (which may be done at outside labs within 2 months of the screening visit)
  - Hematology: CBC, differential, platelets
  - Serum Chemistries: including calcium, magnesium, SGOT, SGPT, alkaline phosphatase, LDH, total bilirubin, BUN, creatinine, electrolytes, and glucose
- Baseline questionnaires

### 8.3 Psilocybin session

- Blood pressure at start of session and 1 hour after administration. If the 1 hour BP is elevated above 140/90 mm Hg, the BP will be repeated hourly until it returns to baseline, and then every 2 hours until 6 hours after psilocybin administration.

- Labs to be done at start of session
    - Urine pregnancy test for participants of childbearing potential
- At the end of the medication dosing session:
    - Participants will complete paper or computer-based questionnaires designed to assess acute subjective experiences associated with the medication dosing session within 24 hours of completing the session.
    - Study facilitators will assess the participant's mood and suicidality with the C-SSRS if indicated.
    - Participants will remain under observation by the facilitation and research team until the facilitation team judges that their perception, cognition, functioning, and judgment are adequate for them to go to bed safely.
- Criteria for booster dose safety check (60-90 min after initial dose psilocybin):
    - Participant must be able to walk without assistance (although a facilitator will be walking along side) to the evaluation station in the room
    - Blood pressure must be BP <u><150 mm Hg systolic or <90 mm Hg diastolic</u>
    - Temperature must be <100.4° F (38° C)
    - No evidence of serotonin syndrome using Hunters criteria
    - Participant reports low subjective sense of drug effect
    - QTc <450 ms on 12 lead ECG

### 8.4 Integration visit safety checks

- Facilitators will be alert to participant symptoms of anxiety or depression on integration visit safety checks and will reach out after the group session to any participant who may need additional support and assessment. An announcement will be made at the beginning of every session inviting any participant to send an individual chat message to the facilitator or to text the study coordinator if any concerns related to suicidality are arising. The study coordinator will then perform an C-SSRS assessment and contact the PI for further assessment.

### 8.5 Post-Treatment Follow-up Period (specify visits based on days after psilocybin session)

Subjects will be seen in group integration sessions on zoom. Their questionnaires will be timed to coincide with these group integration sessions.

--Integration sessions are as follows:

Group Integration 1 is in-person, on Day 3 of the retreat
Individual Integration 1 is in-person, on Day 3 of the retreat
Group Integration 2 is 1 week after leaving the retreat (virtual)
Group Integration 3 is 3 weeks after leaving the retreat (virtual)
Group Integration 4 is 5 weeks after leaving the retreat (virtual)

--Any participant who appears to display any warning signs of a worsening mood (more withdrawn, more hopeless, more helpless in any session will be contacted immediately after a session for an assessment including C-SSRS suicidality assessment. That C-SSRS would be done on a private phone or video call.

--Online questionnaires will be completed after the psilocybin session on the days of the Group Integration visits.

Once these visits and assessments have been completed, the subject will be considered as having completed participation in the clinical trial. If a subject enrolls in another study or requires additional cancer treatment, the visit or assessment dates may be adjusted to accommodate the subject's schedule.

If a subject is unable/unwilling to return for any scheduled visits after the end of trial (EOT), the subject will be considered as having completed study requirements at the EOT.

### 8.6 Long-Term Follow-up Period (specify duration and timing)

Subjects will be contacted by site staff by telephone, email, or mail at 6 months after the psilocybin session to complete scheduled study questionnaires.

### 8.7 Plan of Action for A Participant Exhibiting Suicidality

***For suicidality exhibited on a video call (preparation session or integration session):***

--the study PI (a physician with training in assessing suicidality) or co-investigator (a PhD psychologist also trained to assess suicidality) will immediately call the participant individually, and will conduct a narrative assessment of suicidal wish, method, intent, and plan according to the FDA guidelines on assessing suicidality in clinical trials involving an IND. The assessment will also include administration of the C-SSRS, the instrument approved by the FDA for assessing suicidality in IND clinical trials.

--for a participant with active suicidal ideation (which may include method, or intent, or plan), the study PI will immediately consult the Attending Psychiatrist in the Harborview Emergency Room, who is available 24 hours/day to discuss the case.

--based on the Attending Psychiatrist's recommendations, the study PI may ask the participant to come to the Harborview Emergency Room immediately. If the participant lives a long distance from Harborview Medical Center, the study PI will contact the participant's medical provider (which the PI secures during the consent process) to find an appropriate local referral.

***For suicidality exhibited at the retreat:***

--the study PI (a physician with training in assessing suicidality) or co-investigator (a PhD psychologist also trained to assess suicidality) will immediately assess the participant individually in person, and will conduct a narrative assessment of suicidal wish, method, intent, and plan according to the FDA guidelines on assessing suicidality in clinical trials involving an IND (attached). The assessment will also include administration of the C-SSRS, the instrument approved by the FDA for assessing suicidality in IND clinical trials.

--for a participant with active suicidal ideation (which may include method, or intent, or plan), the study PI will immediately consult the Attending Psychiatrist in the Harborview Emergency Room, who is available 24 hours/day to discuss the case.

--If the Attending Psychiatrist recommends that the patient be seen in an Emergency Room, the study PI will discuss whether the best plan would be for the participant to be transported to the Harborview Emergency Room (a 2 hour drive) or to be seen at a local Emergency Room (within 15 min of the retreat center).

## 9.0 TOXICITY MONITORING

Both acute and chronic toxicities are recorded. Monitoring for acute toxicity takes place during and immediately following IP administration for a period of 8 hours at the study site. Note that a 12-lead ECG with immediate interpretation by a remote cardiologist is available on-site. Subjects are observed for the development of an immediate localized allergic reaction or anaphylactic reaction or emotional reaction during this time.

A rare reported complication of psilocybin use is Hallucinogen Persisting Perceptual Disorder (HPPD), which results in visual perceptual distortions that are generally mild (this is sometimes described as the visual equivalent of tinnitus). This has not been reported in a recent clinical trial but this condition has been reported in anecdotes in the lay literature. Because this is a rare condition and there is no clear treatment, subjects will be warned about this in the consent process, reminded after the psilocybin session, and assessments will be individualized to the subject's symptoms.

There are no known unacceptable toxicity treatments. However, it should be noted that during the psilocybin session that emotional distress or fear or even paranoia is best treated first with facilitator attention and calming,

with medical treatments like benzodiazepines or antipsychotics used as a last resort. In recent clinical trials with investigators at New York University and Johns Hopkins, these 'rescue' medications have never been used (personal communications, A. Back).

## 10.0 SUBJECT DISCONTINUATION OF ACTIVE TREATMENT

Subjects may be removed from this study at any time at their discretion *except* during the psilocybin session. Subjects may also be removed from this protocol if they develop any untoward side effects from the study medications. In addition, there are stopping rules in place for lack of efficacy and excessive toxicity as detailed in the statistical section.

If a subject withdraws consent to participate in the study or aspects of the study, attempts should be made to obtain permission to record survival data up to the protocol-described end of the subject follow-up period. Survival data are important to the integrity of the final study analysis. Documentation in the medical record should state that the subject is withdrawing from the study and what, if any, selected data the subject will permit the investigator to obtain.

During the psilocybin session, the subject will be able to withdraw from the study once the study facilitators and PI have assessed the subject and determined that they are safe for discharge from the session.

Follow up for subjects who withdraw will be dependent on their willingness to be in contact with the PI, but the PI will offer informal follow up at the subject's convenience in order to detect and report adverse outcomes.

Subjects who withdraw will likely not be replaced unless they withdraw before the psilocybin session at a time when a new subject could be recruited to join the small group prior to the first Preparation session.

An explanation for discontinuing treatment is recorded for each subject discontinuing treatment on the appropriate CRF/eCRF. The Sponsor, or its designee, must be notified immediately if a subject discontinues treatment. All subjects, irrespective of treatment status, will continue to be followed for survival. Treatment in this study must be discontinued for any of the following reasons:

- if the Sponsor decides to stop the study;
- at Investigator's discretion;
- at the subject's request;
- if the subject enrolls in a trial of another investigational agent;
- Grade 4 or life-threatening toxicity (See Section 12, Adverse Events) attributable to study agent;
- toxicity reactions of Grade 3 or higher, according to the grading in Appendix XX - NAME Grading Scale as identified in Section 10
- pregnancy;
- any mental health emergent or urgent condition.

## 11.0 CONCOMITANT MEDICATIONS

- Subjects may take small amounts of topical or inhaled corticosteroids, as well as corticosteroids used as replacement therapy for adrenal steroids, e.g., <0.75 mg of dexamethasone, or equivalent.
- Subjects may take doses of nonprescription strength NSAIDS, acetaminophen (paracetamol), ibuprofen or acetylsalicylic acid (aspirin) for non-chronic headache, muscle pain, trauma, or prophylaxis as long the dosing regimens comply with the recommended dose in the product labeling.
- Subjects may receive antihistamine therapy for colds or allergies at non-prescription doses, but subjects should not take these medications within 5 days before or after IP administration.
- Subjects may take vitamin supplements within a dose range not associated with toxicity.
- The use of other investigational agents is not permitted.

## 12.0 ADVERSE EVENTS

### 12.1 Adverse Event

An adverse event (AE) is any untoward medical occurrence in a clinical investigation subject administered a medicinal product; the event does not necessarily have a causal relationship with study drug administration or usage. An adverse event can therefore be any unfavorable and unintended sign (including an abnormal laboratory finding), symptom, or disease temporally associated with the use of a medicinal (investigational) product, whether or not considered related to the medicinal (investigational) product.

Because psilocybin is a Schedule 1 drug, this study will capture and record abuse-related adverse events. The list of abuse-related AEs (Medical Dictionary for Regulatory Activities Preferred Terms (MedDRA PTs) recommended by the FDA includes:

1. Abnormal Behavior
2. Affect Lability
3. Aggression
4. Agitation
5. Amnesia
6. Anxiety
7. Bradyphrenia
8. CSSR abnormal
9. Confusional state
10. Delirium
11. Delusion, Delusion of grandeur, delusional perception, mixed delusion
12. Depersonalization
13. Depression
14. Derealisation
15. Disinhibition
16. Disorientation
17. Disturbance in attention
18. Drug abuse, Drug abuser, Substance abuser, Substance abuse, Drug use disorder, Drug use disorder, antepartum Drug use disorder, postpartum, Substance use disorder
19. Drug diversion
20. Drug dependence, Dependence, Drug dependence, antepartum, Drug dependence, postpartum, Substance dependence
21. Drug withdrawal syndrome, Drug withdrawal convulsions, Drug withdrawal headache, Drug withdrawal maintenance therapy, Withdrawal arrhythmia, Withdrawal catatonia, Withdrawal hypertension, Withdrawal syndrome
22. Dysphoria
23. Elevated mood
24. Emotional disorder
25. Energy increased
26. Euphoria
27. Euphoric mood
28. Feeling abnormal
29. Feeling drunk
30. Feeling jittery
31. Feeling of relaxation
32. Flight of ideas

33. Hallucination, Hypnagogic hallucination, Hypnopompic hallucination, Hallucination auditory, Hallucination gustatory, Hallucination olfactory, Hallucination synesthetic, Hallucination tactile, Hallucination visual, Hallucinations mixed
34. Homicidal ideation
35. Hypersomnia, Hypersomnia related to another mental condition
36. Hypervigilance
37. Inappropriate affect
38. Intentional overdose
39. Irritability
40. Logorrhea
41. Mania
42. Memory impairment
43. Mental disorder
44. Mood altered, Mood disorder due to a general medical condition, Substance-induced mood disorder
45. Mood swings
46. Paranoia
47. Psychomotor hyperactivity
48. Psychotic disorder, Acute psychosis, Transient psychosis, Substance-induced psychotic disorder
49. Restlessness
50. Sedation, Infant sedation, Post-injection delirium sedation syndrome, Sedation complication, Sedative therapy
51. Sensory disturbance
52. Somnolence
53. Stupor
54. Suicidal ideation
55. Thinking abnormal
56. Intentional misuse of drug delivery system, Intentional product misuse, Prescription form tampering, Product tampering, Suspected product tampering

**12.2 Serious Adverse Event**

An AE is classified as a serious adverse event (SAE) if it meets one of the following criteria:

| | |
|---|---|
| Fatal | The AE resulted in death. |
| Life threatening | The AE placed the subject at immediate risk of death. This classification does not apply to an adverse event that hypothetically might cause death if it were more severe. |
| Hospitalization | The AE required or prolonged inpatient hospitalization. Hospitalizations for elective medical or surgical procedures or treatments planned before enrollment in the treatment plan or routine check-ups are not SAEs by this criterion. Admission to a palliative unit or hospice care facility is not considered to be a hospitalization. |
| Disabling/incapacitating | The AE resulted in a substantial and persistent disruption of the subject’s ability to carry out normal life functions. |
| Congenital anomaly or birth defect | An adverse outcome in a child or fetus of a subject exposed to the molecule or treatment plan regimen before conception or during pregnancy. |
| Medically significant | The AE did not meet any of the above criteria, but could have jeopardized the subject and might have required medical or surgical intervention to |

| | |
|---|---|
| | prevent one of the outcomes listed above. |

### 12.3 Unexpected Adverse Event

An unexpected adverse event is defined as an event that has a nature or severity, or frequency that is not consistent with the applicable investigator brochure. "Unexpected," as used in this definition, refers to an adverse drug experience that has not been previously observed and reported rather than an experience that has not been anticipated based on the pharmacological properties of the study drug.

### 12.4 Monitoring and Recording Adverse Events

All AEs will be assessed by the investigator or qualified designee and recorded in the CRFs. The investigator should attempt to establish a diagnosis of the event on the basis of signs, symptoms and/or other clinical information. In such cases, the diagnosis should be documented as the adverse event and/or serious adverse event and not described as the individual signs or symptoms. The following information should be recorded:

- Description of the adverse event using concise medical terminology
- Description as to whether or not the adverse event is serious, noting all criteria that apply
- The start date (date of adverse event onset)
- The stop date (date of adverse event resolution)
- The severity (grade) of the adverse event
- A description of the potential relatedness of the adverse event to study drug, a study procedure, or other causality
- The action taken due to the adverse event
- The outcome of the adverse event

Subjects who terminate early or who experience a non-serious AE considered to be possibly or definitely related to the investigational study agent will be contacted at the time intervals specified in the study for questionnaire assessments with an email asking them how they are doing. If the AE has persisted or has created complications, the PI will request a phone call to hear a more detailed report of the AE, including the information in the above list.

### 12.5 Grading Adverse Event Severity

All AEs will be graded in severity but this study will not use the NCI Common Terminology Criteria for Adverse Events (CTCAE) Version 5.0 because that grading scale is designed for anticancer agents. For this study we will use the following grading system that was approved by the FDA:
--Mild adverse event (does not affect patient activity)
--Moderate adverse event (mild disruption in usual activity)
--Severe (major disruption in usual activity)

### 12.6 Attribution of an Adverse Event

Association or relatedness to the study agent will be assessed by the investigator as follows:

| | |
|---|---|
| **Definite (must have all 4)** | • Has a reasonable temporal relationship to the intervention<br>• Could not have readily been produced by the participant's clinical state or have been due to environmental or other interventions<br>• Follows a known pattern of response to intervention<br>• Disappears or decreases with reduction in dose or cessation of intervention and recurs with re-exposure |

| | |
|---|---|
| **Probable (must have 3)** | • Has a reasonable temporal relationship to the intervention<br>• Could not have readily been produced by the participant's clinical state or have been due to environmental or other interventions<br>• Follows a known pattern of response to intervention<br>• Disappears or decreases with reduction in dose or cessation of intervention |
| **Possible (must have 2)** | • Has a reasonable temporal relationship to the intervention<br>• Could not have readily been produced by the participant's clinical state<br>• Could not readily have been due to environmental or other interventions<br>• Follows a known pattern of response to intervention |
| **Unlikely (must have 2)** | • Does not have a temporal relationship to the intervention<br>• Could readily have been produced by the participant's clinical state<br>• Could have been due to environmental or other interventions<br>• Does not follow a known pattern of response to intervention<br>• Does not reappear or worsen with reintroduction of intervention |

For general AE assessment, an AE is considered related if it is assessed as definitely, probably, or possibly related; unrelated if it is assessed as unlikely related or unrelated.

### 12.7 Adverse Event Recording Period

AEs will be monitored and recorded in study-specific case report forms (CRFs) from the time of first exposure to a study-related intervention in this study, through the date of the last questionnaire at 24 weeks. AEs with an onset date prior to the first exposure to a study intervention will not be recorded, except in the case of clinically significant worsening of the AE during the specified AE monitoring time frame.

A new procedure in this study compared to the prior study BACK002 is that adverse events will be collected using a structured form on the day of the psilocybin session (at the end of the session), 24 hours later, and 48 hours later, to capture any AEs that could be related to the higher dose.

### 12.8 . Adverse Event Reporting Requirements

#### 12.8.1 Reporting to the IRB

The investigator or designee must report events to the IRB of record in accordance with the policies of the IRB for expedited reporting and continuing review.

#### 12.8.2 Reporting to the Coordinating Center [and/or Lead Investigator]

There are no external clinical sites. Thus the PI will be collecting all reports of AEs directly and an additional procedure is not required.

#### 12.8.3 Reporting to FDA

The sponsor-investigator assumes responsibility for IND safety reporting to the FDA and participating investigators, in accordance with regulations under 21 CFR 312.32.

The sponsor-investigator will assess each reported event for seriousness, expectedness, and the relationship to the investigational product.

For determination of IND safety reporting, AE attribution will be assessed according to the suspected adverse reaction definition in 21 CFR 312.32, as an AE for which there is a reasonable possibility that the drug caused the adverse event, where “reasonable possibility” means there is evidence to suggest a causal relationship between the drug and the AE. Suspected adverse reactions that are serious and unexpected will be reported to the FDA as an IND safety report.

SAEs that do not meet IND Safety Report criteria will be reported to the FDA as part of annual reporting responsibilities described under 21 CFR 312.33.

#### 12.8.4 Reporting to Psilo Scientific Ltd

Psilo Scientific Ltd is providing the investigational product for use in the study. All SAEs occurring during the AE reporting period must be reported to Psilo Scientific within 5 days of study team awareness by the study PI.

## 13.0 CRITERIA FOR ENDPOINT EVALUATIONS

All the endpoint evaluations in this study are self-reported questionnaires addressing mental health symptoms, quality of life, and other symptoms. All the evaluation questionnaires are scientifically validated, are listed in the Schedule of Evaluations (Appendix A) and are included in the IRO application.

## 14.0 ASSESSMENT OF EFFICACY

### 14.1 Efficacy and endpoint assessment

Note that the primary endpoint in this study is an adverse event, and efficacy is the secondary endpoint.

Primary Endpoint: Occurrence of any adverse event graded Severe on the Common Terminology Criteria for Adverse Events (CTCAE) commonly used in cancer clinical trials.

The secondary endpoint is the measurement of symptoms of depression and anxiety using the Hospital and Anxiety Depression Scale-Anxiety score (HADS) at 28 days (4 weeks) after the psilocybin session.

### 14.2 Method and Timing

Primary endpoint: At the end of the psilocybin session, adverse effects will be assessed using CTCAE questionnaires by trained study investigators. At all subsequent timepoints, adverse effects will be assessed using CTCAE patient-reported questionnaires.

Secondary and exploratory endpoint: Questionnaires that will be completed online according to the study schedule in Appendix A.

## 15.0 STATISTICAL CONSIDERATIONS

### 15.1 Study Design

This is a single arm, interventional, Phase 1 study with up to 16 participants.

### 15.2 Objectives and Hypotheses

**Primary Objective**: To test the safety of a second group psilocybin retreat for patients with metastatic cancer who participated in BACK002, as measured using patient-reported instruments delivered on a mobile phone app.

Hypothesis:. A second psilocybin-assisted therapy experience, including a booster dose, will be safe, and not associated with any severe adverse events.

**Secondary Objective**: Symptoms of depression and anxiety using the Hospital Anxiety and Depression Scale (HADS) at 1 week, 3 weeks, 5 weeks, 8 weeks, 12 weeks, and 6 months post psilocybin-assisted group therapy.

Hypothesis: The small group model of psilocybin-assisted therapy with demonstrated safety will also demonstrate efficacy, defined as improvement in the Hospital Anxiety and Depression Scale—Total score (HADS-Total) that is comparable to historical data from studies using an individual model of psilocybin-assisted therapy.

### 15.3 Primary/Secondary Endpoints/Hypotheses and Analytical Methods

***Primary outcome analyses.*** Descriptive statistics will be used to analyze all adverse events, and in particular severe adverse events.

***Efficacy analyses of measures of anxiety and other symptoms*.** A pre-post analytic strategy will be used, with the primary measure (HADS) compared at Day -14 (where the medication day = Day 0) and Day 28. We expect that we will see in improvement in the mean HADS value (day 28 vs. day -14), where "improvement" is meant as a mean change in HADS of less than zero. There is no comparison group in this trial, and for each group size, we'll be estimating the "effect size" of change in HADS, where effect size is defined as the mean change divided by the standard deviation of change. To calculate standard deviation, we shall ignore the potential "group" effect and consider all individuals within an intervention group as independent from each other as well as independent from individuals from other intervention groups. While we are hoping to see changes in HADS that are at least comparable to previous studies and this assessment will be descriptive, we will formally test the null hypothesis that the mean change in HADS is statistically different from the fixed value of 0.

### 15.4 Randomization

All subjects in this study will be receiving psilocybin. A future study could compare group psilocybin-assisted therapy to another treatment using randomization.

### 15.5 Exploratory Analysis

Descriptive statistics will be used for all outcomes.

### 15.6. Process measures

Use of these process measures is exploratory in this small sample, and will be analyzed descriptively.

## 16.0 DATA MANAGEMENT/CONFIDENTIALITY

### 16.1 Data Type

**A. Types and amount of scientific data expected to be generated in the project:**

This study will provide data from scientifically valid questionnaires and a qualitative interview completed by study subjects who provide ratings and descriptions of their symptoms, and experience with psilocybin-assisted group therapy. In addition, this study will collect data on blood pressure, subject distress, and subject adverse events during the psilocybin session.

A complete list of the questionnaires is in Appendix A.

**B. Scientific data that will be preserved and shared, and the rationale for doing so:**

The raw questionnaire data, interview data, and blood pressure, distress and adverse event data, and data for administrative linkages will be stored within a secure computing environment. All direct respondent identifiers (e.g., names and addresses) will be removed and maintained in a secure file for future contact purposes.

If this scientific data is shared in the future (no plans exist at this time, subject identifiers will not be shared.

**C. Metadata, other relevant data, and associated documentation:**
Documentation to be made publicly available to the research community will include a list of the questionnaires (which are publicly available), study procedures, qualitative codebook, and research procedures (such as components of the screening evaluation), as well as study-level meta-data.

**16.2 Related Tools, Software and/or Code:**
Scientific data will be processed and analyzed with SAS, SPSS, and Excel, as well as Dedoose for qualitative data.

**16.3 Standards**
To facilitate data use, the study will use standard processing and documentation protocols adopted by the Inter-university Consortium for Political and Social Research (ICPSR) for data formats and dictionaries as well as for variable names, descriptions, and labels.

Demographic, economic, and relationship questions will be based on NCI standards.

**16.4 Data Preservation, Access, and Associated Timelines**
**A. Repository where scientific data and metadata will be archived:**
Data and metadata will be archived on secure UW research servers. Access is restricted to authorized study staff.

**B. Who will receive what records, where, and when.**
Only authorized study staff will have access to identifier files, study databases, and CRFs. CRFs that are completed by hand during psilocybin sessions will be stored in a locked cabinet in the PI's office, and will be transferred to electronic data files stored on secure UW servers designed for research data.

**C. Protections for privacy, rights, and confidentiality of human research participants:**
Subjects' identifying information is only accessed by approved staff as part of the project duties within a secure computing environment. All questionnaire data will be identified only by a study code during collection and analysis.

The privacy, rights, and confidentiality of human subject participants in this study will be protected through the suppression of all direct respondent identifiers, the careful classification of any potentially identifying data as restricted access, and we have applied for a Certificate of Confidentiality.

**16.5 Oversight of Data Management and Sharing:**
Monitoring of and compliance with this Data Management and Sharing Plan will be the responsibility of the project's Principal Investigator. The plan will be implemented and managed by professional staff working under the direction of the PI.

## 17.0 DATA AND SAFETY MONITORING PLAN

Institutional support of trial monitoring will be in accordance with the Fred Hutch/UW/SC Cancer Consortium Institutional Data and Safety Monitoring Plan (DSMP). Under the provisions of this plan, Fred Hutch Clinical Research Support (CRS) coordinates data and compliance monitoring conducted by consultants, contract research organizations, or Fred Hutch employees unaffiliated with the conduct of the study. Independent monitoring visits occur at specified intervals determined by the assessed risk level of the study and the findings of previous visits per the institutional DSMP.

In addition, protocols are reviewed at least annually and as needed by the Consortium Data and Safety Monitoring Committee (DSMC), Fred Hutch Scientific Review Committee (SRC) and the Fred Hutch Institutional Review Board (IRB). The review committees evaluate accrual, adverse events, stopping rules, and adherence to the applicable data and safety monitoring plan for studies actively enrolling or treating subjects. The IRB reviews the study progress and safety information to assess continued acceptability of the risk-benefit ratio for human subjects. Approval of committees as applicable is necessary to continue the study.

The trial will comply with the standard guidelines set forth by these committees and other institutional, state, and federal guidelines.

## 18.0 INVESTIGATOR OBLIGATIONS

The PI is responsible for the conduct of the clinical trial at the site and oversight of the treatment of all study subjects. The PI must assure that all study site personnel, including sub-Investigators and other study staff members, adhere to the study protocol and to all applicable regulations and guidelines regarding clinical trials both during and after study completion.

The PI must ensure that all subjects are informed of the nature of the program, its possible hazards, and their right to withdraw at any time, and each subject signs a form indicating their consent to participate prior to undergoing any study-related procedures.

## 19.0 ADMINISTRATIVE AND REGULATORY CONSIDERATIONS

### 19.1 Pre-Study Documentation

The following documentation required by the FDA must be received by the IND Sponsor, or its designee, prior to initiation of the trial: FDA Form 1572; curricula vitae of the PI and all Sub-Investigators; signed Protocol Agreement; copy of the correspondence from the IRB indicating approval of the protocol and Informed Consent Forms, signed by the IRB chairperson or designee; an IRB membership list containing the names and occupations of the IRB members; copy of the Informed Consent Forms that were reviewed and approved by the IRB.

### 19.2 Study Site Training

Before initiation of the study, the IND Sponsor or its designated representatives will review and discuss the following items with the Investigator and clinic staff: the protocol, study procedures, record keeping and administrative requirements, drug accountability, AE reporting, Good Clinical Practice guidelines, CRF/eCRF completion guidelines, monitoring requirements, and the ability of the site to satisfactorily complete the protocol. Additional documents with instructions for study compliance and CRF/eCRF completion will be provided.

## 19.3 Documentation

The documentation of clinical data must be stored by the IND Sponsor according to legal requirements. The PI and study staff are responsible for maintaining a comprehensive and centralized filing system containing all study-related documentation. These files must be suitable for inspection by the IND Sponsor (if applicable), the FDA, and/or other applicable regulatory agencies at any time, and should consist of the following elements: subject files (complete medical records, laboratory data, supporting source documentation, and the Informed Consent); study files (the protocol with all amendments, copies of all pre-study documentation, and all correspondence between the FDA (as needed), IRB, site, and IND Sponsor (if applicable)); and drug accountability files, containing a complete account of the receipt and disposition of the study drug.

## 19.4 Access to Source Data

The PI will permit the Cancer Consortium representatives or, if applicable, IND Sponsor's representatives, to monitor the study as frequently as deemed necessary to determine that protocol adherence and data recording are satisfactory. The CRF/eCRF and related source documents will be reviewed in detail at each site visit. Only original source documents are acceptable for review. This review includes inspection of data acquired as a requirement for participation in this study and other medical records as required to confirm information contained in the CRF/eCRF, such as past history, secondary diagnoses, and concomitant medications. Other study records, such as correspondence with the IND Sponsor, IRB, and other committees, as well as  screening and drug accountability logs will also be inspected. All source data and study records must also be available for inspection by representatives of the FDA or other regulatory agencies.

## 19.5 Data Collection

Electronic case report forms must be completed and submitted for each subject enrolled in the study. Any changes or corrections made to the CRF/eCRF must be subsequently reviewed and signed by the PI. All data fields in the CRF/eCRF must be completed to avoid queries.

## 19.6 Protocol Interpretation and Compliance

The procedures defined in the protocol are carefully reviewed by the PI and his/her staff prior to the time of study initiation to ensure accurate representation and implementation. Protocol amendments, if any, are reviewed and implemented promptly following IRB approval and FDA authorization to proceed (if applicable). The IND Sponsor is responsible for submitting protocol amendments to the FDA as described in 21 CFR § 312.30 (Protocol Amendments) and other regulatory agencies according to national, state, or local requirements.

For this study, the IND Sponsor-Investigator, or its designee, is always available to answer protocol- or subject-related questions.

## 19.7 Study Monitoring and Data Collection

The IND sponsor-investigator will monitor adherence to the protocol, applicable FDA regulations and/or other regulatory agencies national, state, or local requirements, and the maintenance of adequate and accurate clinical records. Electronic case report forms are reviewed to ensure that key safety and efficacy data are collected and recorded as specified by the protocol. The IND Sponsor or its designee is permitted to access source documentation as needed to appropriately monitor the trial.

## 19.8 Disclosure of Data/Publication

Individual subject medical information obtained as a result of this study is considered confidential and disclosure to third parties other than those noted below is prohibited. Such medical information may be given to the subject's personal physician or to other appropriate medical personnel responsible for the subject's welfare. Data generated as a result of this study are to be available for inspection on request by the FDA or other regulatory agencies, and by the IRB.

### 19.9 Ethical Considerations

The Investigator agrees to conduct this study in accordance with applicable United States FDA clinical trial regulations and guidelines, applicable United States FDA clinical trial regulations and guidelines, the ICH E6 (R2) GCP guidelines, the European Union Directive 2001/20/EC for clinical trials conducted in the European Union, the IRB and local legal requirements and with the Declaration of Helsinki (1989). The Investigator will conduct all aspects of this study in accordance with all national, state, and local laws of the applicable regulatory agencies.

### 19.10 Informed Consent

The PI assumes the responsibility of obtaining written Informed Consent for each subject or the subject's legally authorized representative before any study-specific procedures are performed.

Subjects meeting the criteria set forth in the protocol will be offered the opportunity to participate in the study. To avoid introduction of bias, the Investigator must exercise no selectivity with regard to offering eligible subjects the opportunity to participate in the study. Subjects or parents/legal guardians of all candidate subjects will receive a comprehensive explanation of the proposed treatment, including the nature of the therapy, alternative therapies available, any known previously experienced adverse reactions, the investigational status of the study drug, and other factors that are part of obtaining a proper Informed Consent. Subjects will be given the opportunity to ask questions concerning the study, and adequate time to consider their decision to or not to participate.

Informed Consent will be documented by the use of a written Consent Form that includes all the elements required by FDA regulations and ICH guidelines. The IND Sponsor or designee will review the informed consent prior to submission to the IRB. The form is to be signed and dated by the subject or subject's legally authorized representative and by the person who administers the consent process. A copy of the signed form will be given to the person who signed it, the original signed Consent Form will be filed with the subject's medical records, and copy maintained with the subject's study records. The date and time of time of the Informed Consent must be recorded in the source documents.

If an amendment to the protocol changes the subject participation schedule in scope or activity, or increases the potential risk to the subject, the Informed Consent Form must be amended. Any amended Informed Consent must be reviewed by the IND Sponsor or designee (if applicable) and approved by the IRB prior to use. The revised Informed Consent Form must be used to obtain re-consent from any subjects currently enrolled in the study if the subject is affected by the amendment, and must be used to document consent from any new subjects enrolled after the approval date of the amendment.

### 19.11 Institutional Review Board

The PI will assure that an appropriately constituted IRB that complies with the requirements of 21 CFR Section 56 or written assurance of compliance with ICH E6(R2) guidelines will be responsible for the initial and continuing review and approval of the clinical study. Before initiation of the study, the PI or designee will forward copies of the protocol and Consent Form to be used for the study to the IRB for its review and approval.

The PI or designee will also assure that all changes in the research activity and all unanticipated problems involving risks to human subjects or others will be reported promptly to the IRB, and that no changes will be made to the protocol without prior IND Sponsor (if applicable) and IRB approval, except where necessary to eliminate apparent immediate hazards to human subjects.

If the study is under an IND, copies of relevant study-related correspondence between the Investigator and the IRB may be provided to the IND Sponsor, or its designee, by the Investigator. The PI or designee must promptly notify the IRB of any SAE occurring at the site or of any safety reports received from the IND Sponsor or designee according to the requirements of the IRB of record.  The Investigator or designee will be responsible for submitting

periodic progress reports to the IRB at intervals appropriate to the degree of subject risk involved in the study, but not less than once per year and at the completion or termination of the study.

### 19.12 Subject Privacy

The IND Sponsor and the Investigator affirm and uphold the principle of the subject's right to privacy. The IND Sponsor, its designees, and the Investigator shall comply with applicable national and local privacy laws.

To verify compliance with this protocol, the IND Sponsor or designee will require that the Investigator permit the IND Sponsor or designee's monitor to review the subject's original medical records. Should access to such medical records require a waiver or authorization separate from the statement of Informed Consent, the Investigator will obtain such permission in writing from the subject before the subject is entered into the study.

### 19.13 Controlled Substance Issues

Psilocybin is a Schedule I (CI) substance under the Controlled Substances Act (CSA; 21 CFR 1308.11). Activities associated with your proposed investigation under this IND must comply with the applicable Drug Enforcement Administration (DEA) regulations for research, manufacturing, handling, and storage requirements for a Schedule I drug (21 CFR Part 1301). A Schedule I investigator license issued by the DEA is required prior to the initiation of a study with psilocybin and is issued or updated for each specific study protocol and amended protocol.

The importation of psilocybin for this study from Filament Health/Psilo Scientific Ltd in Canada will be done according to DEA regulatory procedures for importation of a Schedule I drug.

The unauthorized use of the psilocybin by individuals other than the subjects (e.g., by study staff) will be reported. Any instances of drug accountability discrepancies that may have occurred at the study site will be pursued with identification of the origin of the discrepancy and individuals involved.

## 20.0 STOPPING THE STUDY

The Sponsor-Investigator may decide to stop the study at any point, for any reason. The following reasons will lead to premature termination of the trial:

- New convincing information leading to unfavorable risk-benefit assessment of the investigational agent, including occurrence of significant toxicity associated with the investigational agent, psilocybin, being used at a higher dose than in the prior study.
- Sponsor-investigator's decision that continuation of the trial is unjustifiable for medical or ethical reasons.
- Subject-level stopping criteria are as follows:
    - The booster dose described in Section XX will NOT be offered:
        - If hypertension with BP >150 mm Hg systolic or >90 mm Hg diastolic; or
        - If clinical suspicion of serotonin syndrome using the Hunter criteria is detected; or
        - If the QTc is >450 ms.
- Study-level stopping criteria are as follows:
    - There are 2 retreats with up to 8 participants per retreat planned in this protocol. The study will be stopped and the second retreat will NOT be held if the following conditions are met:
        - BP >170 mm Hg systolic or BP>110 diastolic is experienced by 2 or more participants
        - Anxiety or other psychological disturbance requiring 1:1 facilitator attention for >30 min is experienced by 4 or more participants simultaneously
        - Paranoid ideation even if transient is experienced by 2 or more participants.
          These parameters would represent a considerable increase in risk compared to published studies (Ross et al) and our unpublished experience from BACK002.

## 21.0 REFERENCES


1. Ross S, Bossis A, Guss J, et al. Rapid and sustained symptom reduction following psilocybin treatment for anxiety and depression in patients with life-threatening cancer: a randomized controlled trial. J Psychopharmacol 2016;30(12):1165-1180. DOI: 10.1177/0269881116675512.
2. Griffiths RR, Johnson MW, Carducci MA, et al. Psilocybin produces substantial and sustained decreases in depression and anxiety in patients with life-threatening cancer: A randomized double-blind trial. J Psychopharmacol 2016;30(12):1181-1197. DOI: 10.1177/0269881116675513.
3. Grob CS, Danforth AL, Chopra GS, et al. Pilot study of psilocybin treatment for anxiety in patients with advanced-stage cancer. Arch Gen Psychiatry 2011;68(1):71-8. DOI: 10.1001/archgenpsychiatry.2010.116.
4. Niedzwiedz CL, Knifton L, Robb KA, Katikireddi SV, Smith DJ. Depression and anxiety among people living with and beyond cancer: a growing clinical and research priority. BMC Cancer 2019;19(1):943. DOI: 10.1186/s12885-019-6181-4.
5. Ayubi E, Bashirian S, Khazaei S. Depression and Anxiety Among Patients with Cancer During COVID-19 Pandemic: A Systematic Review and Meta-analysis. J Gastrointest Cancer 2021;52(2):499-507. DOI: 10.1007/s12029-021-00643-9.
6. Shaw J, Pearce A, Lopez AL, Price MA. Clinical anxiety disorders in the context of cancer: A scoping review of impact on resource use and healthcare costs. Eur J Cancer Care (Engl) 2018;27(5):e12893. DOI: 10.1111/ecc.12893.
7. Soleimani MA, Bahrami N, Allen KA, Alimoradi Z. Death anxiety in patients with cancer: A systematic review and meta-analysis. Eur J Oncol Nurs 2020;48:101803. DOI: 10.1016/j.ejon.2020.101803.
8. Grossman CH, Brooker J, Michael N, Kissane D. Death anxiety interventions in patients with advanced cancer: A systematic review. Palliat Med 2018;32(1):172-184. DOI: 10.1177/0269216317722123.
9. Passie T, Seifert J, Schneider U, Emrich HM. The pharmacology of psilocybin. Addict Biol 2002;7(4):357-64. (In eng). DOI: 10.1080/1355621021000005937.
10. Nichols DE. Psychedelics. Pharmacol Rev 2016;68(2):264-355. DOI: 10.1124/pr.115.011478.
11. Usdin E, Efron DH. Psychotropic Drugs and Related Compounds. Washington D.C.: National Institute of Mental Health, 1972.
12. COMPASS Pathways and King's College London Announce Results From Psilocybin Study In Healthy Volunteers. 2019.
13. Franz M, Regele H, Kirchmair M, et al. Magic mushrooms: hope for a 'cheap high' resulting in end-stage renal failure. Nephrol Dial Transplant 1996;11(11):2324-27. DOI: 10.1093/oxfordjournals.ndt.a027160.
14. Lim TH, Wasywich CA, Ruygrok PN. A fatal case of 'magic mushroom' ingestion in a heart transplant recipient. Intern Med J 2012;42(11):1268-9. DOI: 10.1111/j.1445-5994.2012.02955.x.
15. Gerault A, Picart D. Intoxication mortelle à la suite de la consommation volontaire et en groupe de champignons hallucinogeèes. Bull Soc Mycol France 1996;112:1-14.
16. Buck RW. Mushroom Toxins — A Brief Review of the Literature. New Engl J Med 1961;265:681-686.
17. van Amsterdam J, Opperhuizen A, van den Brink W. Harm potential of magic mushroom use: a review. Regul Toxicol Pharmacol 2011;59(3):423-9. DOI: 10.1016/j.yrtph.2011.01.006.
18. Tyls F, Palenicek T, Horacek J. Psilocybin--summary of knowledge and new perspectives. Eur Neuropsychopharmacol 2014;24(3):342-56. DOI: 10.1016/j.euroneuro.2013.12.006.
19. Studerus E, Kometer M, Hasler F, Vollenweider FX. Acute, subacute and long-term subjective effects of psilocybin in healthy humans: a pooled analysis of experimental studies. J Psychopharmacol 2011;25(11):1434-52. DOI: 10.1177/0269881110382466.
20. Carbonaro TM, Bradstreet MP, Barrett FS, et al. Survey study of challenging experiences after ingesting psilocybin mushrooms: Acute and enduring positive and negative consequences. J Psychopharmacol 2016;30(12):1268-1278. DOI: 10.1177/0269881116662634.
21. Johnson M, Richards W, Griffiths R. Human hallucinogen research: guidelines for safety. J Psychopharmacol 2008;22(6):603-20. (In eng). DOI: 10.1177/0269881108093587.

22. Johnson MW, Griffiths RR, Hendricks PS, Henningfield JE. The abuse potential of medical psilocybin according to the 8 factors of the Controlled Substances Act. Neuropharmacology 2018;142:143-166. (In eng). DOI: 10.1016/j.neuropharm.2018.05.012.
23. Treatment Episode Data Set (TEDS) 1994-2004. DEPARTMENT OF HEALTH AND HUMAN SERVICESSubstance Abuse and Mental Health Services AdministrationOffice of Applied Studies 2006. (https://wwwdasis.samhsa.gov/dasis2/teds_pubs/2004_teds_rpt.pdf).
24. Swift TC, Belser AB, Agin-Liebes GI, et al. Cancer at the Dinner Table: Experiences of Psilocybin-Assisted Psychotherapy for the Treatment of Cancer-Related Distress. Journal of Humanistic Psychology 2017;57(5):488-519.
25. Agin-Liebes GI, Malone T, Yalch MM, et al. Long-term follow-up of psilocybin-assisted psychotherapy for psychiatric and existential distress in patients with life-threatening cancer. J Psychopharmacol 2020;34(2):155-166. DOI: 10.1177/0269881119897615.
26. Erritzoe D, Barba T, Spriggs MJ, Rosas FE, Nutt DJ, Carhart-Harris R. Effects of discontinuation of serotonergic antidepressants prior to psilocybin therapy versus escitalopram for major depression. J Psychopharmacol 2024;38(5):458-470. (In eng). DOI: 10.1177/02698811241237870.
27. Goodwin GM, Aaronson ST, Alvarez O, et al. Single-Dose Psilocybin for a Treatment-Resistant Episode of Major Depression. N Engl J Med 2022;387(18):1637-1648. (In eng). DOI: 10.1056/NEJMoa2206443.
28. Barbut Siva J, Barba T, Kettner H, et al. Interactions between classic psychedelics and serotonergic antidepressants: Effects on the acute psychedelic subjective experience, well-being and depressive symptoms from a prospective survey study. J Psychopharmacol 2024;38(2):145-155. (In eng). DOI: 10.1177/02698811231224217.
29. Hasler F, Grimberg U, Benz MA, Huber T, Vollenweider FX. Acute psychological and physiological effects of psilocybin in healthy humans: a double-blind, placebo-controlled dose-effect study. Psychopharmacology (Berl) 2004;172(2):145-56. (In eng). DOI: 10.1007/s00213-003-1640-6.
30. Moreno FA, Wiegand CB, Taitano EK, Delgado PL. Safety, tolerability, and efficacy of psilocybin in 9 patients with obsessive-compulsive disorder. J Clin Psychiatry 2006;67(11):1735-40. (In eng). DOI: 10.4088/jcp.v67n1110.
31. Garcia-Romeu A, Barrett FS, Carbonaro TM, Johnson MW, Griffiths RR. Optimal dosing for psilocybin pharmacotherapy: Considering weight-adjusted and fixed dosing approaches. J Psychopharmacol 2021;35(4):353-361. DOI: 10.1177/0269881121991822.
32. Bogenschutz MP, Ross S, Bhatt S, et al. Percentage of Heavy Drinking Days Following Psilocybin-Assisted Psychotherapy vs Placebo in the Treatment of Adult Patients With Alcohol Use Disorder: A Randomized Clinical Trial. JAMA Psychiatry 2022;79(10):953-962. (In eng). DOI: 10.1001/jamapsychiatry.2022.2096.
33. O'Donnell KC, Mennenga SE, Owens LT, et al. Psilocybin for alcohol use disorder: Rationale and design considerations for a randomized controlled trial. Contemp Clin Trials 2022;123:106976. (In eng). DOI: 10.1016/j.cct.2022.106976.
34. Brown RT, Nicholas CR, Cozzi NV, et al. Pharmacokinetics of Escalating Doses of Oral Psilocybin in Healthy Adults. Clin Pharmacokinet 2017;56(12):1543-1554. (In eng). DOI: 10.1007/s40262-017-0540-6.
35. Vollenweider FX, Kometer M. The neurobiology of psychedelic drugs: implications for the treatment of mood disorders. Nat Rev Neurosci 2010;11(9):642-51. (In eng). DOI: 10.1038/nrn2884.
36. Nicholas CR, Henriquez KM, Gassman MC, et al. High dose psilocybin is associated with positive subjective effects in healthy volunteers. J Psychopharmacol 2018;32(7):770-778. (In eng). DOI: 10.1177/0269881118780713.
37. Greenstein M, Breitbart W. Cancer and the experience of meaning: a group psychotherapy program for people with cancer. Am J Psychother 2000;54(4):486-500. DOI: 10.1176/appi.psychotherapy.2000.54.4.486.
38. Leszcz M, Goodwin PJ. The rationale and foundations of group psychotherapy for women with metastatic breast cancer. Int J Group Psychother 1998;48(2):245-73. DOI: 10.1080/00207284.1998.11491538.
39. Mikkelsen N, Damkier P, Pedersen SA. Serotonin syndrome-A focused review. Basic Clin Pharmacol Toxicol 2023;133(2):124-129. (In eng). DOI: 10.1111/bcpt.13912.

## 22.0 APPENDICES

Appendix A: Study Calendar
Appendix B: ECOG Performance Status Scale

## Appendix A Study Schedule

| Schedule for Activities For A Participant | | | | | | | | | |
|---|---|---|---|---|---|---|---|---|---|
| **Purpose** | **Screen visit** | **Group Prep #1** | **Group Prep #2** | **Group Prep #3** | **Medi-cation** | **Integra-tion #1** | **Integra-tion #2** | **Integra-tion #3** | **Integra-tion #4** |
| **Study day** | **-15+** | **-14** | **-7** | **-1** | **0** | **1** | **8** | **22** | **36** |
| Visit number | **V0** | **V1** | **V2** | **V3** | **V4** | **V5** | **V6** | **V7** | **V8** |
| **Research activity** | | | | | | | | | |
| Demographics | X | | | | | | | | |
| Eligibility Determination | X | | | | | | | | |
| Medical exam | X | | | | | | | | |
| Informed consent | X | | | | | | | | |
| Caregiver contact info | X | | | | | | | | |
| Labs, ECG, urine tox review | X | | | | | | | | |
| Pregnancy screen (if applicable) | X | | | | X | | | | |
| PI review of inclusion/exclusion | X | | | | | | | | |
| Psychiatric evaluation | X | | | | | | | | |
| Education re expectations | X | X | | | | | | | |
| Preparation Session (group) | | X | X | X | | | | | |
| Preparation Session (individual) | | | | X | | | | | |
| Medication (psilocybin) | | | | | X | | | | |
| Medication dosing monitoring | | | | | X | | | | |
| Integration psychotherapy | | | | | | X | X | X | |
| Adverse event reporting | | X | X | X | X | X | X | X | X |
| | | | | | | | | | |
| **Research activity** | | | | | | | | | |
| **Measures** | | | | | | | | | |
| Hosp Anxiety Depr Scale (HADS) | X | X | | | | | X | | X |
| FACT-G | | X | | | | | X | | X |
| Adjustment Disorder Questionnaire | | X | | | | | X | | X |
| Watts Connectedness Scale | | X | | | | | X | | X |
| NIH-HEALS | | X | | | | | X | | X |
| Demoralization II (DS-II) | | X | | | | | X | | X |
| Death and Dying Distress Scale | | X | | | | | X | | X |
| Suicidality (C-SSRS)* | X | X | X | X | X | X | X | | |
| Mystical Experience Questionnaire** | | | | | X | | | | |
| Emotional Breakthrough Inventory** | | | | | X | | | | |
| Challenging Experiences Q** | | | | | X | | | | |
| Communitas Scale** | | | | | X | | | | |
| Psychedelic Music Questionnaire** | | | | | X | | | | |
| Strength of Drug Effect | | | | | X | | | | |
| Group identification scale -Cohesion | | | | X | | | X | | X |
| Social identification | | | | X | | | X | | X |
| Social support | | | | X | | | X | | X |
| Purpose and Meaning Scale | | | | X | | | X | | X |
| Global impression of Change Scale | | | | X | | | X | | X |
| Qualitative interview*** | | | | | | | | | X |
| Adverse events | X | X | X | X | X | X | X | X | X |

Notes on following page:

***Note that the C-SSRS, a clinician-administered instrument that is required at the screening visit. Visit 3 (the day before the psilocybin session), and Visit 5 (the day after the psilocybin session. At other visit, the C-SSRS will be done at the discretion of the facilitators and will require an individual call (it will not be administered during a group visit).**
****The time window for completing the V4 questionnaires is 24 hours.**
*****The qualitative interview will be done during a time window starting after V8 up to V9.**

| Follow up measures for participants after integration sessions end | | | |
|---|---|---|---|
| **Purpose** | **Follow up** | **Follow up** | **Follow up** |
| **Study Day** | 56 (8 wk) | 84 (12 wk) | 168 (24 wk) |
| **Visit number** | **V9** | **V10** | **V11** |
| **Research activity** | | | |
| **Measures** | | | |
| Hosp Anxiety Depr Scale (HADS) | X | X | X |
| FACT-G | X | X | X |
| Adjustment Disorder Questionnaire | X | X | X |
| Watts Connectedness Scale | X | X | X |
| NIH-HEALS | X | X | X |
| Demoralization II (DS-II) | X | X | X |
| Death and Dying Distress Scale | X | X | X |
| Group Identification Scale – Cohesion | X | X | X |
| Social identification item | X | X | X |
| Social support | X | X | X |
| Purpose and Meaning | X | X | X |
| Global impression of change | X | X | X |
| Adverse events | X | X | X |

Please note that these “Visits” are not actually in-person or virtual visits but are questionnaire followups only, but we are calling them “Visits” for the purpose of data tracking and analysis.

*Note that the C-SSRS will not be done at Visits 9, 10, and 11 because those Visits do not involve clinician contact.

**Appendix B ECOG Performance Scale**

| GRADE | SCALE |
|---|---|
| 0 | Fully active, able to carry out all pre-disease performance without restriction |
| 1 | Restricted in physically strenuous activity but ambulatory and able to carry out work of a light or sedentary nature, e.g., light housework, office work |
| 2 | Ambulatory and capable of all self-care but unable to carry out work activities. Up and about more than 50% of waking hours. |
| 3 | Capable of only limited self-care, confined to bed or chair more than 50% of waking hours. |
| 4 | Completely disabled. Cannot carry on any self-care. Totally confined to bed or chair. |
| 5 | Dead |
