# Supplementary material for: A phase 1 study of a second experience with Group Retreat Psilocybin Therapy for partial responders after a first experience
Source: Front Public Health. 2026 Apr 14;14:1810904. doi: 10.3389/fpubh.2026.1810904 (PMC13122983; doi:10.3389/fpubh.2026.1810904)
Supplement: Supplementary file 2 [file Data_Sheet_2.pdf]

Reprinted with permission from Pronovost-Morgan et al., licensed under CC BY 4.0, https://doi.org/10.1038/s41591-025-03685-9

ReSPCT Guidelines 2025 – Reporting Table for BACK004 **13 Feb 2026**
Nat Med. 2025.

Item	Item Number	Item details	Reported on page number:
**Physical environment**			
Location	1	Location of the trial, and whether indoors/outdoors and urban/rural/suburban.	The Whidbey Institute, a nature-based retreat center 2 hours from Seattle; inside Thomas Berry Hall
Ambiance	2	Room ambiance curated by the study team.	ustic, secular, a communal altar was assembled
Access to nature	3	Sources of nature or natural elements that are physically or visually accessible to participants.	Thomas Berry Hall has a wall of windows that open to large trees, a meadow, and rolling trails
Objects and decorations	4	Objects and decorations in the room.	The room is decorated with a large painting of the Earth, and simple wood furnishings.
Lighting	5	Room lighting and adjustability.	Room lighting is mostly natural from skylights, with minimal ambient light that can be adjusted
Sensory reduction	6	Sensory reduction used, such as headphones and eyeshades.	Headphones and eyeshades were offered; Speakers were playing the same music simultaneously.
Bathroom facilities	7	Level of bathroom accessibility and privacy.	The bathrooms are accessible and private, located just outside the front door of the hall.
**Dosing session procedure**			
Number and roles of people present	8	Number and roles of people present, including participants, study staff and informal support.	4 facilitators, 2 assistant facilitators, 2 study assistants.
Positioning	9	Relative position of people in the room, and what participants were positioned on (e.g., bed, mat, couch).	Chairs (for Day 1 and 3) or the Mattresses were arrayed in a circle around the altar in the center of the room
Focus and main activities	10	Focus (internal or external) and main activities of the dosing session.	The focus for the dosing session was both individual (stating intentions and going inward) and communal (witnessing others, an opening and closing circle)
Music or soundscapes	11	Music or soundscapes that accompanied the dosing experience.	Continuous music created by Wavepaths for this study,
Interpersonal interventions	12	Verbal or physical interpersonal interventions used throughout the session, and how consent was obtained.	Consent was obtained prior to arrival at the retreat center, and repeated in a private meeting on Day 1. Touch guidelines have been published. Verbal interventions were minimal unless the participant

			was dysregulated and distressed. A private room was available nearby.
Participant autonomy, control, and agency	13	Level of participant control and agency over activities and environment of the dosing session.	Participants were able to choose whether to use headphones, to sit up or lie down or walk around in the room or outside (accompanied).
Dosing regimen	14	Dosing regimen, including drug dose(s), frequency, route of administration and length of the dosing session.	A single dose of psilocybin 35 mg was given. At 60-90 min, a booster dose of 10 mg was available after a safety check. The dosing session lasted about 7 hours..
Medical and experimental procedures or assessments	15	Medical and experimental procedures or assessments performed during the dosing session.	Blood pressure at the beginning, and an hour later; followed hourly as long as BP was elevated. The booster safety check included a brief neurologic exam to assess for serotonin syndrome, temperature, BP; and a drug effect 2 item instrument was given.
Pre- and post-dosing protocol	16	Activities that took place immediately prior to- or postdosing, including participant arrival and release conditions.	Described in Methods.
Potential disturbances or interruptions	17	Disturbances or interruptions that may have impacted the quality of the dosing session.	No unexpected disturbances or interrupations occurred.
**Therapeutic framework and protocol**			
Therapeutic or guiding approach	18	Therapeutic/guiding approach used throughout the study, if any, with the accompanying manual or protocol.	See detailed publication on the facilitation model, which is based on rites of passage, reference 4.
Narrative framing	19	Framing of the trial intervention by the study team, including the short- and long-term drug effects.	The trial was framed as offering an inner exploration that would be supported by the facilitators. Short term and long term drug effects were discussed in the screening visit, detailed on the consent form, explained at the retreat Day 1 and as questions arose.
Number of sessions	20	Number and length of preparation, dosing, and integration sessions.	There were 2 virtual prep sessions, and 1 in-person prep session; a single psilocybin session was offered in this trial; 1 in-person integration session; then 3 virtual integration sessions.
Preparation protocol	21	Activities performed during the preparation sessions.	See prior publications, references 3 and 4
Integration protocol	22	Activities performed during the integration sessions.	See prior publication, references 3 and 4
Additional support/follow-up	23	Formal or informal support or follow-up offered to participants after the end of the trial.	Informal support available and offered.
Study personnel qualifications	24	The credentials, training, and expertise of personnel providing the study intervention or care.	All facilitators were licensed clinicians with specialized psychedelic training and trial-specific training. The study coordinator and

			assistant worked on the prior group psilocybin trial and received trial-specific training for this trial.
Cultural competence and safety	25	Study team's level of cultural competence and efforts towards cultural safety.	The facilitator group for each retreat intentionally included a mixture of gender, age, professional background, and race. Cultural issues were discussed at team meetings which occurred twice a day during the retreat.
**Subjective experiences**			
Therapeutic alliance	26	Therapeutic alliance between participants and facilitators throughout the intervention.	We did not measure therapeutic alliance formally. Our informal impression is that participants felt a strong therapeutic alliance with the facilitator team.
Trust	27	Participant's level of trust throughout the intervention.	We did not measure trust formally. Our informal impression is that participants felt a great deal of trust with the facilitator team.
Physical comfort	28	Participant's level of physical comfort during the dosing session.	We did not measure physical comfort formally. Our informal impression is that participants were physically comfortable on Exped mattresses, blankets, and pillows.
Physical safety	29	Participant's sense of physical safety during the dosing session.	We had participants report a high degree of physical safety.
Psychological and cultural safety	30	Participant's sense of interpersonal safety with the people present during the session.	We had participants report a high degree of psychological safety. We did not inquire specifically about cultural safety.
